# Reprogramming of stromal fibroblasts by SNAI2 contributes to tumor desmoplasia and ovarian cancer progression

**DOI:** 10.1186/s12943-017-0732-6

**Published:** 2017-10-17

**Authors:** Zongyuan Yang, Xin Yang, Sen Xu, Ping Jin, Xiaoting Li, Xiao Wei, Dan Liu, Kecheng Huang, Sixiang Long, Ya Wang, Chaoyang Sun, Gang Chen, Junbo Hu, Li Meng, Ding Ma, Qinglei Gao

**Affiliations:** 1 Cancer Biology Research Center (Key laboratory of the ministry of education), Tongji Hospital, Tongji Medical College, Huazhong University of Science and Technology, 1095 Jiefang Anv., Wuhan, Hubei 430030 China; 2 Department of hematology, Tongji Hospital, Tongji Medical College, Huazhong University of Science and Technology, Wuhan, Hubei 430030 China

**Keywords:** SNAI2, Stromal fibroblast, Reprogram, Stiffness, Ovarian cancer

## Abstract

**Background:**

Molecular profiling in ovarian cancer (OC) revealed that the desmoplasia subtype presented the poorest prognosis, highlighting the contribution of stromal fibroblasts in tumor progression. This study aimed to investigate the molecular characteristics of SNAI2 driving the transcriptional reprogramming of fibroblasts within tumors.

**Methods:**

SNAI2 expression was evaluated in microdissected profiles of various cancers and in various molecular subtypes of OC. Gene set enrichment analysis (GSEA) and single sample GSEA (ssGSEA) were performed to explore the correlation between SNAI2 and stromal fibroblast activation. The SNAI2 defined signature in the mesenchymal OC subtype was identified through an integrative analysis of the TCGA and the Tothill datasets. The predictive value of this signature was validated in independent datasets. SNAI2 expression alteration influence of tumor growth in primary CAFs was evaluated in 3D organotypic and murine xenograft models.

**Results:**

We demonstrated that SNAI2 was frequently activated in the tumor stroma, correlated with fibroblast activation and worse patient outcome in OC. SNAI2 transformed normal fibroblasts to a CAF-like state and boosted their tumor–supporting role in 3D organotypic culture and in OC xenograft model. SNAI2 drove a transcriptional signature in the mesenchymal subtype of OC that contributed to tumor desmoplasia, which fed back to increase SNAI2 expression and sustain fibroblast activation.

**Conclusions:**

Our results address the role of SNAI2 in reprogramming stromal fibroblasts. The identified SNAI2 mesenchymal signature has both a predictive value and biological relevance and might be a therapeutic target for stroma-oriented therapy against the desmoplasia OC subtype.

**Electronic supplementary material:**

The online version of this article (doi:10.1186/s12943-017-0732-6) contains supplementary material, which is available to authorized users.

## Background

High grade serous ovarian cancer (OC) remains a devastating gynecological malignancy hallmarked by a high degree of heterogeneity [1]. Conventional classification scheme based on histopathological evaluation failed to achieve the primary objective of directing clinical intervention and stratifying patient prognosis [2]. Inspiringly, tumor molecular profiling has launched a revolution aiming to better discriminate cancer subtypes and decipher the underlying driving molecular events of each category [3]. Up to now, the most successful attempt in this field regarding epithelial OC was conducted by the Australia Ovarian Cancer Study (AOCS) group and The Cancer Genome Atlas (TCGA) network, which unanimously revealed that the subtype with a reactivated tumor stroma presented the worst prognosis [4, 5]. The emerged evolutional conceptions based on molecular profiling are advancing our comprehension of OC heterogeneity and implying the importance of the host stroma components in determining the destiny of OC patients.

Reactivated tumor stroma is closely related with tumor desmoplasia and is characterized by the accretion of reactive stromal cells, extracellular matrix (ECM) and the resultant increased matrix stiffness [6]. The role of desmoplasia in solid tumor initiation and progression is complicated and yet insufficiently characterized [7]. Fibroblasts represent the majority of tumor stromal cells and primarily contribute to the reactivated stroma and tumor desmoplasia [8, 9]. It is generally thought that the transition from normal fibroblasts to an activated state known as cancer-associated fibroblasts (CAFs), under the recruitment and transformation of tumor cells, is fundamental for cancer progression [10, 11]. Even though the reciprocal interactions between cancer cells and fibroblasts has been extensively described, it is still currently unclear how quiescent fibroblasts are reprogrammed into CAFs [12, 13]. In this scenario, a thorough understanding of the regulatory basis underlying fibroblast activation could aid in the development of stromal fibroblast-oriented strategies against the fibrotic subtype of OC.

The deregulation of transcription factors (TFs) is frequently observed in numerous cancer types. Aberrantly activated pathways in tumor cells eventually converge on the activation of sets of TFs to perturb target genes that coordinate tumor progression [14]. To date, TF dysfunction research in cancer has focused on their capacity to initiate the transdifferentiation of epithelial cells, but few studies have dissected the pro-tumorigenic effects of TF expression in the stromal compartment [10]. Recently, TF aberrations were found not merely restricted to tumor cells but also in stromal fibroblasts and lymphocytes [10, 15, 16]. Among them, the importance of SNAI1 expression perturbation in the tumor stroma has been put forward in numerous studies [17]. It was demonstrated to modulate fibroblast activation and ECM remodeling in the tumor stroma and predict poor patient survival in breast and colon cancer [18, 19]. In parallel, Twist1 overexpression was mainly restricted to the tumor stroma and was required for fibroblast activation and matrix stiffness in colorectal cancer [15]. In OC, CAF heterogeneity and the dominant intrinsic TF aberrations are only beginning to be interpreted. Therefore, we are determined to explore the potent transcriptional regulation that is involved in stromal fibroblast activation and the assignment of desmoplasia subtype of OC.

In this study, we demonstrated that SNAI2 was advantageously expressed in stromal fibroblasts and correlated with tumor stromal activation and a worse OC patient prognosis. Mechanistically, SNAI2 defined a signature in the desmoplasia OC subtype, which further contributed to stroma stiffness and subsequent fibroblast activation. SNAI2 transforms normal fibroblasts to an aggressive form and boost their tumor–supporting role in 3D organotypic culture and OC murine xenograft model. Our results address the role of SNAI2 in reprogramming OC stromal fibroblasts and the facilitation of tumor progression. Thus, SNAI2 and its regulated mesenchymal signature could be an attractive target for stroma-oriented therapy against the desmoplasia subtype of OC.

## Methods

### Cell culture

Epithelial OC cell line SKOV3 was purchased from ATCC (Rockville, MD, USA). Fibroblast cell line MRC-5 was obtained from the cell bank of the Chinese Academy of Sciences. All the cell lines were authenticated by their source organizations prior to purchase, routinely checked for mycoplasma contamination and used within 4 months after frozen aliquot recovery. Primary normal ovarian fibroblasts (NFs), ovarian CAFs were purified from EOC patient tumor tissues following procedures as previously described [20]. SKOV3 was maintained in McCoy’s 5A medium and MRC-5, primary NFs and CAFs were cultured in DMEM/F-12 medium with 10% FBS and 1% penicillin/streptomycin (Thermo Scientific), at 37 °C in a 5% CO_2_ and 80% humidity incubator. SKOV3 cells had been stably transduced with CMV-Fluc-IRES-RFP lentiviral particles (GeneChem, shanghai, China) and designated as SKOV3-Luc previously, which were further used in subsequent animal experiments.

### Public database analysis

Gene expression data (GSE40595, GSE38666, GSE14548, GSE9890, GSE14548, GSE35602, GSE9014, GSE39396 profiling data) were downloaded as raw signals from Gene Expression Omnibus (http://www.ncbi.nlm.nih.gov/geo), interpreted, normalized and log2 scaled using the online analysis tool GCBI website (https://www.gcbi.com.cn). The Oncomine expression analysis tool (www.oncomine.org) was used to examine SNAI2 expression in microdissected breast profile GSE9014. Statistical significance is based upon Student’s *t*-test. Gene expression of SNAI2, ACTA2, FAP and PDGFRB in various cancer types was obtained from published gene expression profile included in the TCGA dataset via the cBioPortal tools (http://cbioportal.org). Pearson’s correlation analysis was performed between SNAI2 and classical CAF markers.

### Gene set enrichment analysis (GSEA) and single sample GSEA (ssGSEA)

To determine the enrichment score of specific signatures in the genesets positively correlated with SNAI2 expression in stromal profile GSE40595, GSEA was performed using the publicly available desktop application from the Broad Institute. Gene signatures associated with myofibroblast activation features such as “Stromal_Stimulation_Up”, “Cytoskeleton” and “Smooth_Muscle_Contraction”, and stromal prognostic signatures as “Up_In_Stroma_When_Mets”, “Stromal_Response_Signature” and “Stromal_Signature (Resistance To Chemo)” selected from the MsigDB and published profiles (Additional file 1: Table S1). To compare the activation degree of 141-stroma signature and SNAI2 mesenchymal signature in samples included in OC subtypes, we used ssGSEA to generate geneset activation score as described previously [21].

### Immunohistochemistry and Masson’s trichrome staining

EOC tissues were obtained from patients who had undergone surgery at the Department of Gynecological oncology of Tongji Hospital (Wuhan, China). Informed consent was obtained from all patients. Tumor tissues were from patients diagnosed with advanced (stages III and IV) serous adenocarcinoma. Immunohistochemical staining for SNAI2, was conducted as described in our prior study [22]. Immunostaining scoring of SNAI2 in cellular nucleus of carcinoma cells and fibroblasts of each tissue was assessed using an scoring system encompassing staining intensity (0 = none, 1 = weak, 2 = moderate, 3 = strong) and the proportion of expressing cells (0 = 0%, 1 = 1~25% 2 = 26~50%, 3 = 51~75%, 4 = 76~100%); the sum of the scores produced the final score ranging from 0 to 12. (Score 0 to 4 was defined as low expression and score 5 to 12 was defined as high expression). Associations between SNAI2 expression and clinicopathological characteristics of the patients were analysed with χ2 test (Fisher’s exact test). The prognostic significance of SNAI2 expression and other clinicopathological variables were first assessed by univariate Cox regression analysis using overall survival time in a cohort of 160 EOC patients. Then, multivariate Cox regression analysis was performed to identify the prognostic effect of those factors. Myofibroblast count in xenograft tissues was assessed by the amount of aSMA-positive cells in the medium field of a view. Masson’s trichrome (Sigma, HT15) staining was performed as described previously on paraffin embedded sections of xenograft tumors [23]. Quantification of the matrix content in xenograft tumors was carried out using color thresholding in ImageJ software, including data from 5 to 10 crypt units in 5–10 sections per mouse.

### Western blot analysis and immunofluorescence

Cells were lysed with RIPA lysis buffer (Beyotime, Shanghai, China) supplemented with a protease inhibitor cocktail (Roche). Protein concentration was measured with a bicinchoninic acid (BCA) assay (Thermo Scientific), and 40 μg of total lysate for each sample was subjected to SDS-PAGE followed by blotting with the indicated primary antibodies. Antibodies for SNAI2 (#9585), SNAI1 (#3879) and YAP1 (#14074) were purchased from Cell Signaling Technology (Beverly, MA, USA). Antibodies against ACTA2 (ab5694), FAP (ab28244), PDGFRB (ab32570) and GAPDH (ab9485) were obtained from Abcam Biotechnology (Abcam, CA, USA). Then incubated with HRP-linked secondary antibody (Abcam) and finally detected using an enhanced ECL system (Pierce). Immunofluorescence of the cytoskeleton was performed as conducted previously [24]. F-actin was visualized in cells by incubation with rhodamine-conjugated phalloidin (Thermo Scientific). Fluorescence images were taken using an Olympus BX53 microscope (Olympus, Tokyo, Japan). Quantification of fibroblast cellular area was assayed as total cell area divided by cell amounts and total area in a medium field performed by ImageJ software in each treatment group.

### Survival analysis

To evaluate SNAI2 expression influence of EOC patient outcome, we performed a meta-analysis of 2970 EOC patient expression profiles and generated forest plot using the ‘curatedOvarianData’ Bioconductor package [25]. To generate survival plot for SNAI2 in clinical samples and SNAI2 mesenchymal signature in public datasets, we performed this analysis using the “best cutoff” value of SNAI2 score and signature ssGSEA score (all percentiles are computed and the best performing threshold is automatically chosen as the cutoff). Patients were divided into a high-score and a low-score group using the selected cutoff value. Overall survival curves were calculated using the Kaplan–Meier method, and statistical signifcance was assessed using the log-rank test. The analyses were conducted with the R Bioconductor ‘survival’ package.

### Collagen gel contraction assay

A total of 6 × 10^5^ fibroblasts in each group were suspended in a collagen gel mixture composed of 100 μL of collagen mix (68.75 μl DMEM/F-12 medium, 0.72 μl 1 N NaOH, 31.25 μl Rat Tail Collagen, Type 1) (Thermo) and 100 μL DMEM/F-12 medium per well in a 24-well ultra-low attachment plate (Corning Life Sciences, Corning, NY). Gels were photographed 12 h after fibroblasts planting, and the area was measured using ImageJ software and expressed as a percentage of the original well area. All contraction assays were performed in triple.

### 3D organotypic culture

Three-dimentional organotypic culture was performed as described previously by Ernst Lengyel with some modification [26]. 1 × 10^6^ MRC5 or primary NF cells transfected with either Ade-ctrl or Ade-SNAI2 were added in the bottom layer, and 1 × 10^6^ tumor cells were added to the top layer 3 days after fibroblast implanting. After 10 days of tumor cells implantation, organotypic gels were harvested and embedded in paraffin. Hematoxylin and Eosin (HE) was performed in paraffin sections and images were taken under an Olympus BX53 microscope (Olympus). Quantification of the proliferation assays was performed as described previously using ImageProPlus software [27].

### Transfection of siRNA, adenovirus and lentivirus

MRC5 or primary CAFs were transfected with Lipofectamine 2000 reagent (Invitrogen) using either negative control (si-NC), SNAI1 specific (si-SNAI1), SNAI2 specific (si-SNAI2) or YAP1 specific (si-YAP1) specific siRNAs (Santa Cruz Biotechnology, CA, USA) according to the manufacturer’s protocol. Recombinant adenovirus expressing SNAI2 was obtained from Vigene Biosciences for transient overexpression of SNAI2 in fibroblasts. Lentiviral shRNAs targeting SNAI2 was obtained from Genechem (Shanghai, China) for knocking down of SNAI2 in primary CAFs. RFP-based flow cytometry sorting was performed to select cells stably transduced with lentivirus targeting SNAI2 (sh- SNAI2) or control vector (sh- NC).

### Web-based Gene Set Analysis Toolkit (WebGestalt), STRING and PRECOG analysis

The analysis of gene set enrichment among SNAI2 mesenchymal signature was performed using Web-based Gene Set Analysis Toolkit (WebGestalt) (http://www.webgestalt.org/). STRING (http://stitch.embl.de/) was used to annotate functional interaction of proteins. Survival z-scores for SNAI2 in various cancer types were obtained from the PREdiction of Clinical Outcomes from Genomic Profiles (PRECOG) portal (https://precog.stanford.edu/). Expression of the SNAI2 mesenchymal signature genes mapped on the transcriptome of individual murine hematopoietic and stromal cell types in the ImmGene project (immgen.com). The plot was generated using MyGeneset tool (http://rstats.immgen.org/MyGeneSet/).

### In vitro model of mechanically tunable COL1-coated polyacrylamide (PAA) gel and atomic force microscopy (AFM) measurement

An in vitro system of mechanically tunable COL1-coated polyacrylamide gel was established as previously described [28]. Polyacrylamide gels with different mechanical stiffness levels were prepared by mixing 10% acrylamide and 0.01–0.5% bis-acrylamide (Sigma, USA) in a HEPES-buffered solution (pH = 8) supplemented with 10% ammonium persulfate (1/100 volume) and TEMED (1/100 volume). The following two groups of PA gel substrates were prepared: soft (10% acrylamide/0.05% bis-acrylamide), and stiff (10% acrylamide/0.3% bis-acrylamide). The Young’s moduli of the two gels were measured via indentation experiments using an atomic force microscope (AFM) and found to be 3.42 ± 0.21 kPa (soft), and 26.24 ± 0.42 kPa (stiff), respectively. The formed gels were further crosslinked and coated with 0.1 mg/ml of COL-1solution (BD) to facilitate cell adhesion. Fresh tissue samples preparation for AFM measurements were cut into 1~3 mm sections. Two-component epoxy glue (5 min hardening time) was utilized to be put under the tissue. Then place the sample-mounted round glass onto the sample stage and perform the measurements. SPM9700 scanning probe microscope was used to measure tissue stiffness. The Hertz model was used to determine the elastic properties of the tissue. Tissue samples were assumed to be incompressible and a Poisson’s ratio of 0.5 was used in the calculation of the Young’s elastic modulus.

### Animal assay

The animal study was performed with the approval of the Committee on the Ethics of Animal Experiments in the Hubei province. Four- to Six-week-old female NOD/SCID mice were housed and maintained in laminar flow cabinets under specific pathogen-free condition. SKOV3-Luc (2 × 10^6^) cells were inoculated subcutaneously in right back of the mice, either alone or co-injected with 2 × 10^6^ Ade-NC or Ade-SNAI2 transfected CAFs (SNAI2^low^). In parallel experiment, SKOV3-Luc (2 × 10^6^) cells were inoculated either alone or co-injected with 2 × 10^6^ sh-NC or sh-SNAI2 stably transduced CAFs (SNAI2^high^) (*n* = 8 per group). Approximately 4 weeks later, mice were anaesthetized with 1% pentobarbital sodium, and imaged with the IVIS SPECTRUM system (Caliper, Xenogen, USA). Total flux (photons/s) of xenografts was analyzed using Living Image version 4.3.1 software.

### Statistics

Results are expressed as the means ± standard deviation (s.d.) from at least three independent experiments. Single comparisons between two groups were determined by Student’s t-test. Comparisons between multiple groups were determined by one-way ANOVA followed by Tukey post-test. All statistical analyses were performed in R 3.0.0. *P* values <0.05 were considered significant.

## Results

### SNAI2 is specifically overexpressed in OC stroma and associated with poor survival

**Fig. 1:**
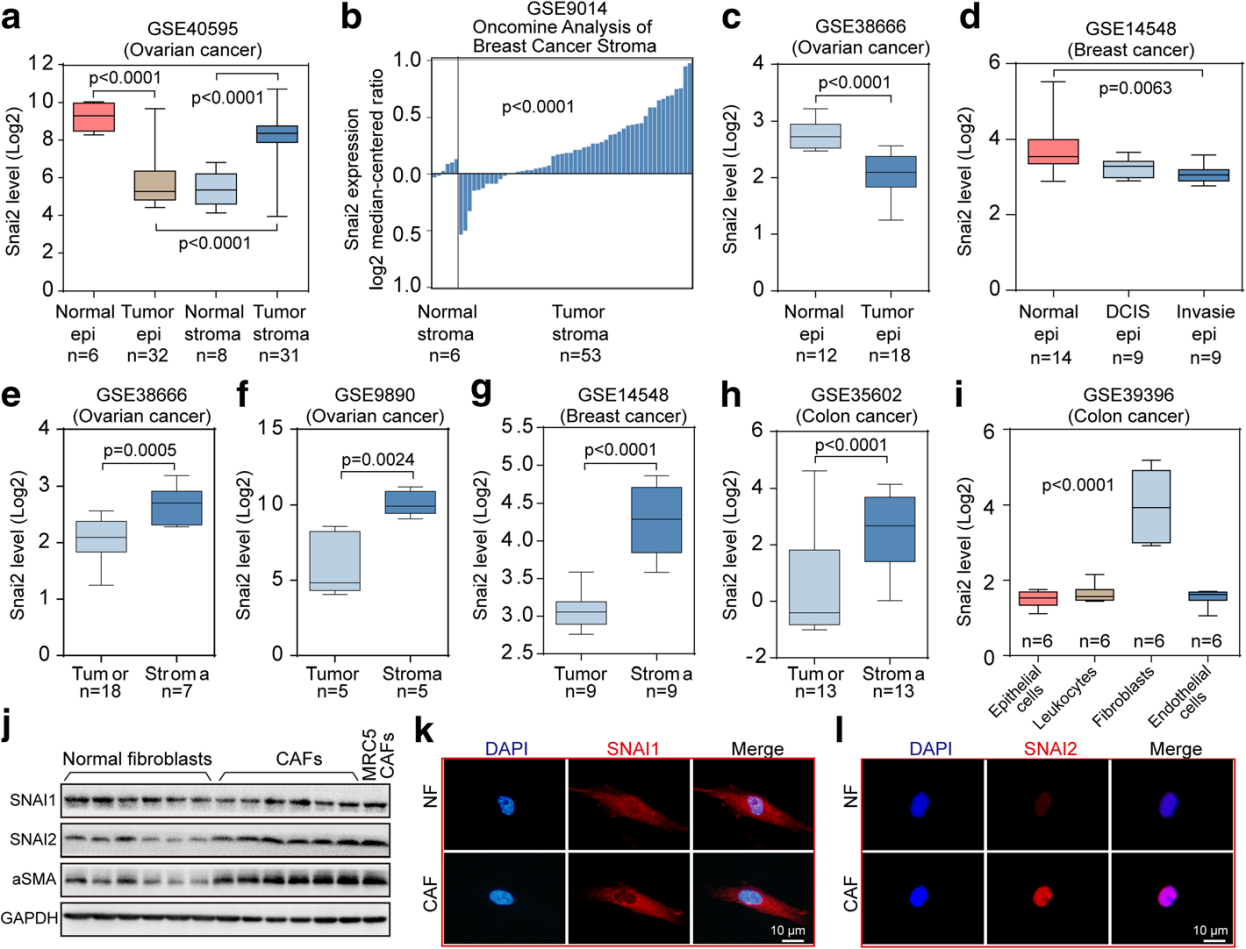
The specific upregulation of SNAI2 in the ovarian tumor stroma. **a** Boxplots showing the expression level of SNAI2 in microdissected normal epithelium, normal stroma, tumor epithelium and tumor stroma included in the ovarian dataset GSE40595. **b** Oncomine analysis of SNAI2 in the microdissected normal and tumor stroma in the breast profile GSE9014. **c** Boxplots showing the expression level of SNAI2 in the microdissected normal and tumor epithelium of GSE38666. **d** Boxplots showing the expression level of SNAI2 in the normal epithelium, the DCIS epithelium and the invasive epithelium of GSE14548. **e–h** Boxplots showing the expression level of SNAI2 in the tumor and stroma compartment in the ovarian dataset GSE38666 (**e**). and GSE9890 (**f**)., the breast dataset GSE14548 (**g**). and the colon dataset GSE35602 (**h**). **i** Illustration of the relative gene expression of SNAI2 in the epithelial cells, leukocytes, endothelial cells, and fibroblasts as deposited in the colon cancer profile GSE39396. **j** Immunoblot showing the expression of SNAI1, SNAI2 and aSMA in the microdissected normal ovarian fibroblasts and the cancer associated fibroblasts from OC patients. MRC5-CAFs served as the positive control for activated fibroblasts. GAPDH served as the loading control. **k–l** Immunofluorescence staining showing the relative expression of SNAI1 (**k**). and SNAI2 (**l**). in the normal ovarian fibroblasts and in the cancer associated fibroblasts from OC patients. The nuclei were counterstained with DAPI. The *P* value is indicated

To explore the expression pattern of SNAI2 in epithelial OC, we analyzed a microdissected profile of OC and found that SNAI2 was decreased in the tumor epithelium and was elevated in the tumor stroma, compared with their normal counterparts (Fig. 1a). The elevation of SNAI2 in stromal cells along tumor progression was validated in microdissected breast profiles (Fig. 1b). In addition, the reduced expression of SNAI2 in epithelial cells along tumor progression was further confirmed in ovarian and breast profiles (Fig. 1c–d). Furthermore, the advantageous expression of SNAI2 in the tumor stroma was next confirmed in another four profiling datasets of ovarian, breast and colon cancer (Fig. 1e–h). In tumor microenvironment, fibroblasts were demonstrated to be the major residents that express SNAI2 (Fig. 1i). The effect of tumoral SNAI2 expression on patient survival was conducted using curatedOvarianData [25]. SNAI2 mRNA was significantly correlated with a poor overall survival (OS) (HR = 1.17, *p* = 1.76e-8) and progression-free survival (PFS) (HR = 1.14, *p* = 2.02e-4) in EOC patients (Additional file 2: Figure S1).

In contrast, the other SNAI family member SNAI1 was expressed in a distinct pattern that was elevated in the epithelial compartment, while no obvious change was found in the stromal tissues compared with their normal counterparts (Additional file 3: Figure S2A). Meanwhile, data mining revealed that a negligible alteration was found between the SNAI1 level in fibroblasts compared with that in epithelial, leukocytes and endothelial cells (Additional file 3: Figure S2A). Accordingly, SNAI1 expression failed to predict prognosis in OC patients (Additional file 3: Figure S2C–D). Finally, immunoblotting and immunofluorescence both revealed that SNAI2 protein level, rather than SNAI1, was particularly elevated in purified ovarian CAFs compared with NFs (Fig. 1j–l).

In addition, SNAI2 was associated with poor survival in most epithelial cancers through analyzing the pan-cancer Genomic Profiles (Additional file 4: Figure S3A) [29]. Associations between the major CAF activation candidate TFs and survival z-scores in several epithelial cancer types revealed that most TFs were associated with a poor survival, with SNAI2 having the strongest association (Additional file 4: Figure S3B). These results suggested that the shift from SNAI1 domination in the tumor epithelium to SNAI2 domination in the tumor stroma might coordinate OC progression.

### SNAI2 expression is associated with a reactive and desmoplastic stroma in OC

**Fig. 2:**
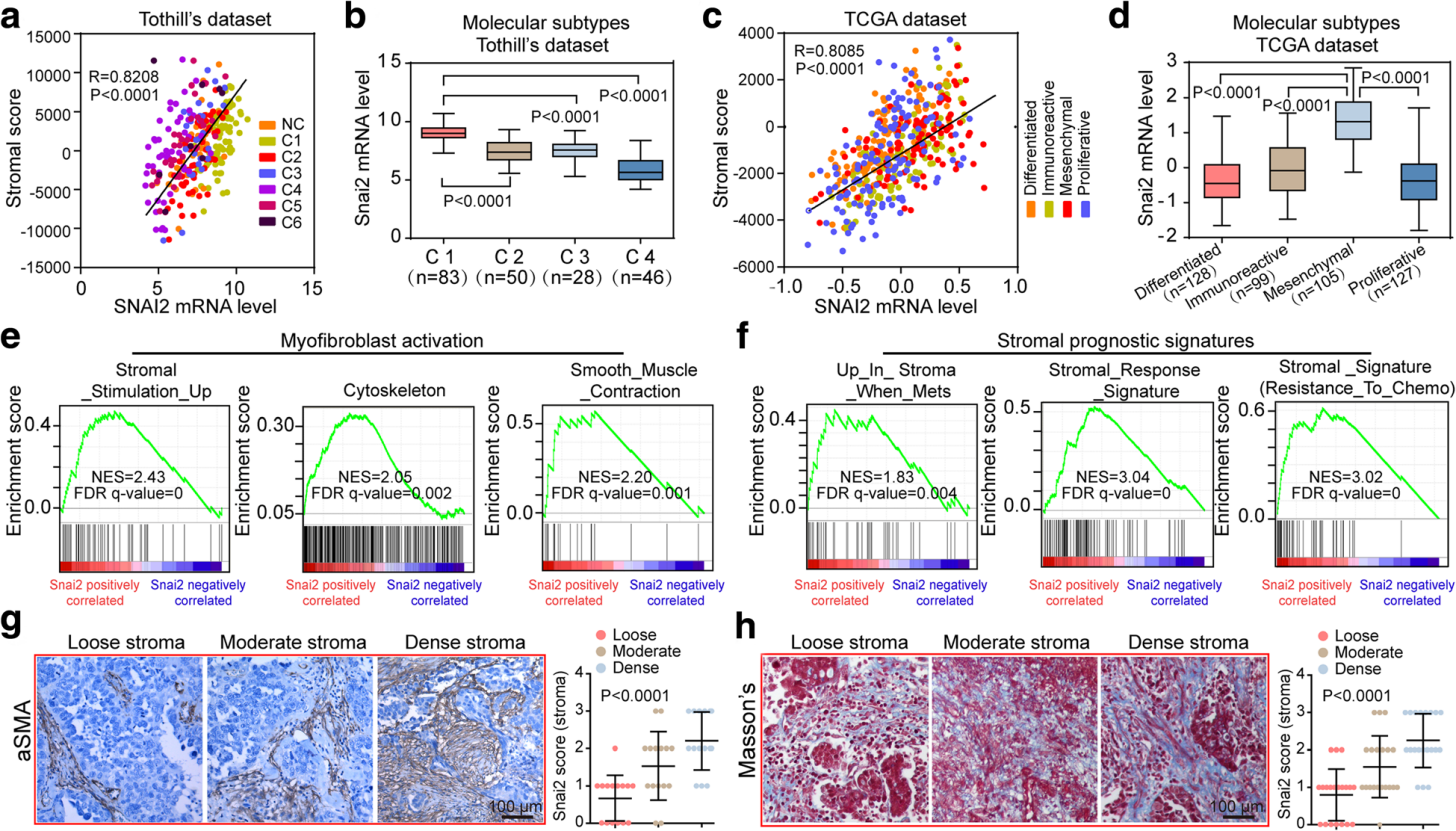
SNAI2 expression is associated with a reactive and desmoplastic stroma in OC. **a** Scatter plots and Spearman’s correlation showing the expression level of SNAI2 and that of the calculated stroma score in the Tothill’s dataset GSE9891. **b** Boxplots showing the relative SNAI2 expression in the major molecular subtypes (C1-C4) of GSE9891. **c** Scatter plots and Spearman’s correlation showing the expression level of SNAI2 and that of the calculated stroma score in the ovarian TCGA dataset. **d** Boxplots showing the relative SNAI2 expression in various subtypes of the ovarian TCGA dataset. **e** and **f** GSEA plot of the association between gene sets positively correlated with SNAI2 in the stroma profile of GSE40595 and the signatures representative of myofibroblast activation (**e**) or the signatures predictive of patient prognosis in the tumor stroma (**f**). **g** and **h** Scoring of SNAI2 in the stromal compartment of OC tissues with variant tumor stroma matrix (Loose stroma, moderate stroma and dense stroma) stratified by the aSMA staining (**g**) and Masson’s staining (**h**). The *P* value is indicated

To identify the biological mechanism that could explain the association between stromal SNAI2 expression and poor survival, we set out to explore whether SNAI2 expression correlates with that of stromal activation. The most classical molecular classification schemes in OC were reported by the AOCS and TCGA networks [4, 5]. Tothill et al. classified the OC dataset into six molecular subtypes (C1-C6), of which the C1 subtype is hallmarked by extensive stromal desmoplasia [4]. Our analysis showed that SNAI2 mRNA was notably correlated with the stromal component and was most highly expressed in the C1 subtype (Fig. 2a–b). In accordance, we discovered that SNAI2 expression was correlated with the stromal component and was most highly expressed in the mesenchymal cluster (Fig. 2c–d), implying that SNAI2 expression was enriched in subtypes with reactive tumor stroma. Moreover, we showed, in TCGA network of variant cancers and the ovarian microdissected profile, that SNAI2 was positively related with classical CAF activation markers, such as ACTA2, FAP, and PDGFRB (Additional files 5 and 6: Figures S4A, C and S5). Besides, SNAI2 was also found positively related with classical ECM remodeling molecules, such as LOX, COL1A1, and SPARC (Additional file 5: Figure S4B, D).

Furthermore, gene set enrichment analysis (GSEA) revealed that SNAI2 expression was positively correlated with signatures representative of myofibroblast activation and stromal prognostic signatures (Fig. 2e–f). To better demonstrate the relationship between stromal snai2 expression and tumor desmoplasia, we stratified OC patients into variant groups assigned as loose, moderate and dense stroma groups according to tissue aSMA or Masson’s trichome staining. Stringkingly, the results showed that stromal SNAI2 expression was most robustly expressed in the aforementioned dense stroma individuals (Fig. 2g–h). These data strongly implied that SNAI2 is a potent TF regulating stromal fibroblast activation that ultimately influence tumor desmoplasia.

### High SNAI2 expression in stromal fibroblasts is associated with worse clinical outcome in OC

**Fig. 3:**
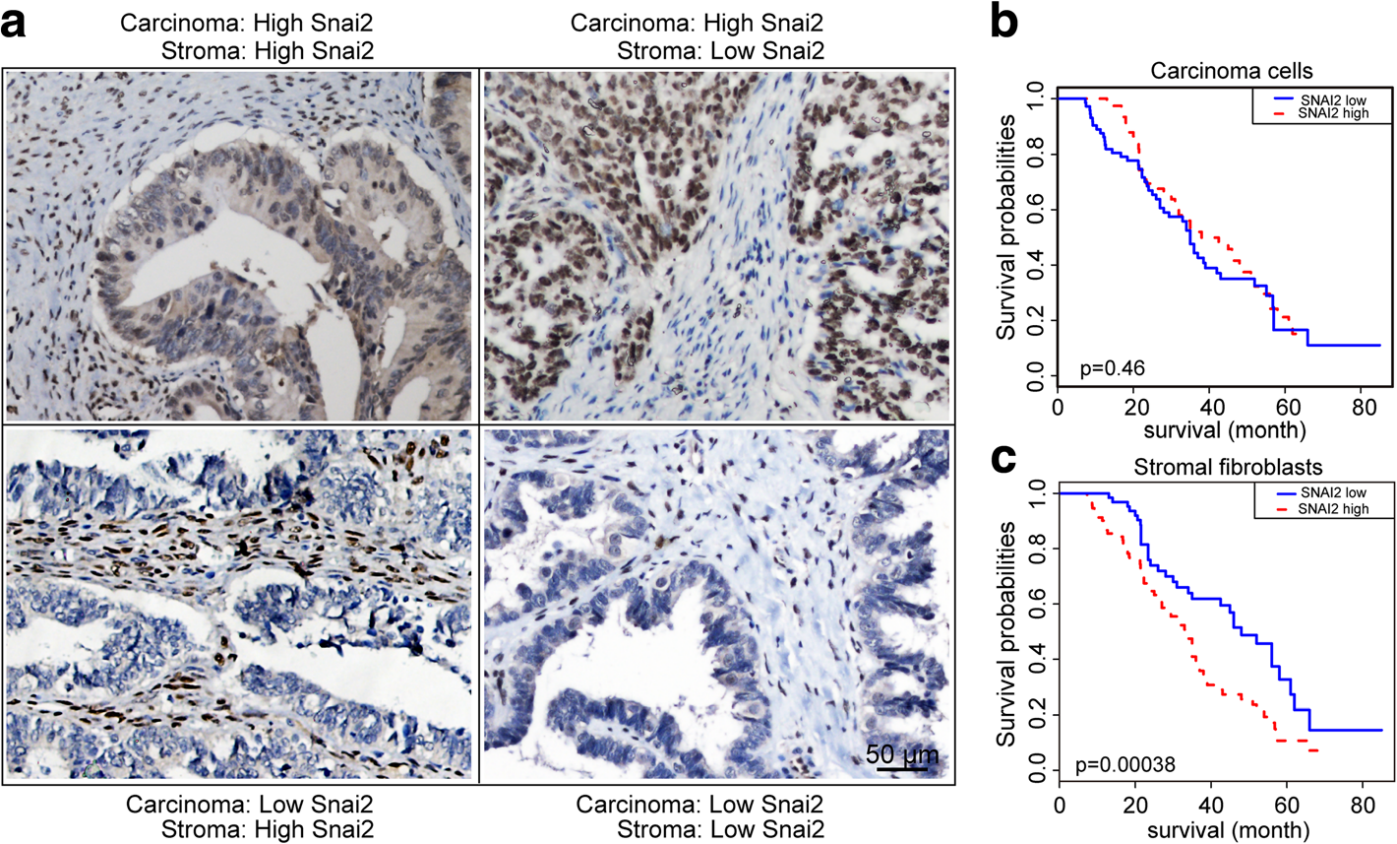
The expression pattern and clinical relevance of tumoral and stromal SNAI2 expression in OC patients. **a** Representative IHC images of the SNAI2 protein expression in the carcinoma and stromal compartments of the OC tumor tissues. **b** and **c** Kaplan–Meier survival curves depicting the overall survival (OS) of patients with serous OC stratified by SNAI2 protein expression levels (relative nuclear staining intensity) in carcinoma cells (**b**) or in tumor stromal fibroblasts (**c**). The *P* value is indicated

Next, the clinical relevance of SNAI2 protein was further investigated in a cohort of 160 serous OC patients with prolonged clinical follow-up (Additional file 1: Table S2). Intense nuclear and faint cytoplasmic SNAI2 expression was detected in carcinoma and stromal cells of human OC. Notably, SNAI2 expression in the stroma and carcinoma compartment of OC tumor tissues differed widely among patients as follow: 21.3% of the tumors showed high SNAI2 levels in both compartments; 18.2% displayed high SNAI2 in the carcinoma cells and low in the stroma; 23.1% presented high SNAI2 in the stroma and low in the carcinoma and 37.4% had low SNAI2 in both compartments (Fig. 3a). Next, we found that SNAI2 in the carcinoma cells exerted a negligible effect in discriminating the patients’ overall survival (OS) (median survival 23.4 vs 30.9 months, *p* = 0.46). Strikingly, high SNAI2 expression in stromal fibroblasts was significantly associated with a decreased OS (median survival 22.3 vs 34 months, *p* = 0.00038) (Fig. 3b–c). Multivariate Cox regression analysis confirmed that SNAI2 expression in stromal fibroblasts was an independent predictor for OS (HR = 2.154, 95% CI 1.393 to 3.331, *p* = 0.0004) (Additional file 1: Table S3).

### SNAI2 transforms normal fibroblasts to a CAF-like state

**Fig. 4:**
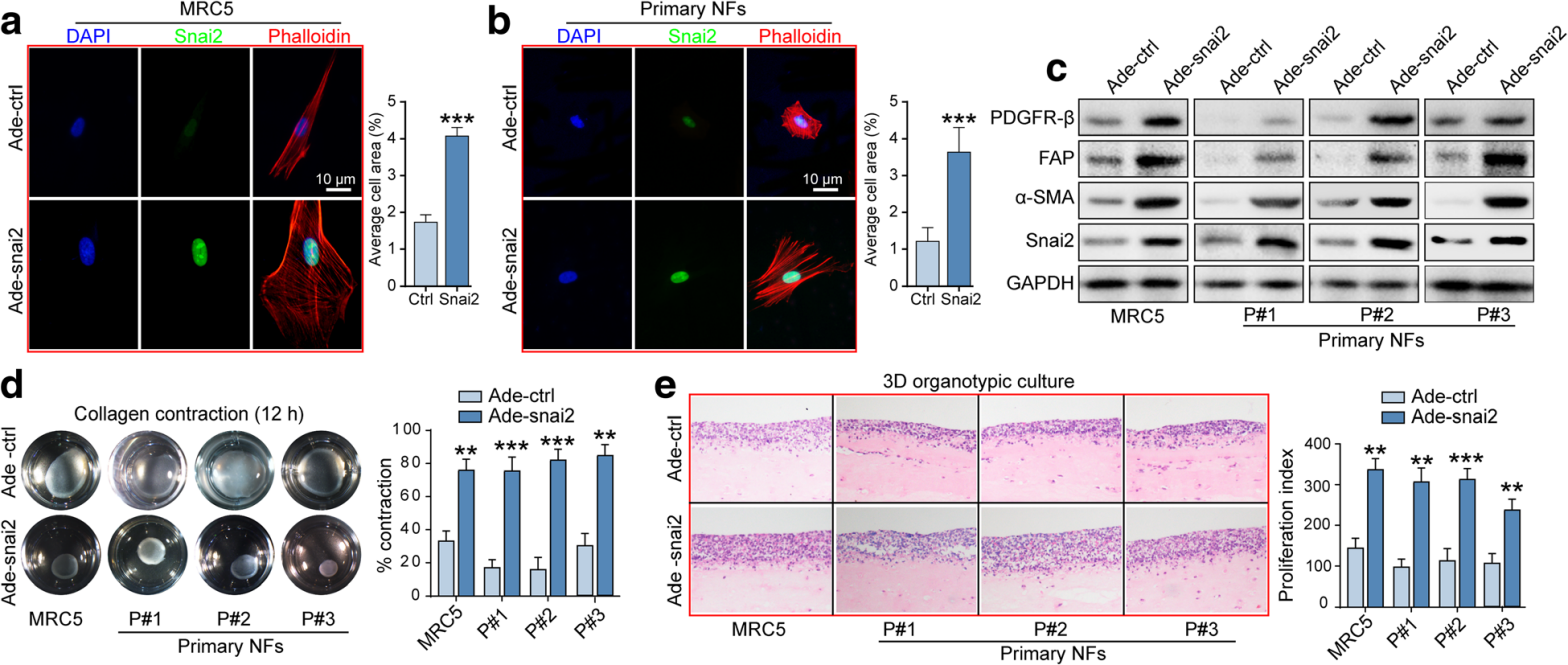
SNAI2 transforms normal fibroblasts to a CAF-like state. **a** and **b** Representative images and quantification of the cellular cytoskeleton by F-actin staining and SNAI2 immunofluorescence in the MRC5 cells (**a**). and the ovarian normal fibroblasts (NFs) (**b**). after transfection with Ade-ctrl or Ade-SNAI2. The nuclei were counterstained with DAPI. **c** Western blot analysis of PDGFRB, FAP, ACTA2 and SNAI2 in the MRC5 cells and the primary ovarian NFs after a 72 h transfection with Adeno-ctrl or Adeno-SNAI2. GAPDH served as the loading control. **d** Representative images and quantification of the collagen contraction capacity of the MRC5 cells and the primary ovarian NFs in the Adeno-ctrl and Adeno-SNAI2 transfection group. **e** Representative images and the quantification of the proliferation index of SKOV3 cells cocultured with MRC5 cells and primary ovarian NFs in the Adeno-ctrl and Adeno-SNAI2 transfection group (**P < 0.01, ***P < 0.001)

Constitutive activation is the basis of stromal fibroblasts that contribute to tumor progression [30]. Toward this end, we examined the effect that SNAI2 overexpression exerts on normal fibroblast activity in several aspects. First, adenovirus mediated SNAI2 overexpression reprogrammed fibroblast cytoskeleton, which was characterized by a cellular shape alteration to a spread spindle appearance under F-actin staining in MRC5 cells and primary normal fibroblasts (Fig. 4a–b). Second, immunoblotting revealed that typical CAF markers, such as FAP, αSMA, and PDGFRB, were notably elevated following the transfection of SNAI2 encoding adenovirus (Fig. 4c). Next, collagen contraction assay also implied that the fibroblasts displayed a remarkably increased capacity in contracting ECM after the acquisition of SNAI2 expression (Fig. 4d). Finally, we constructed a 3-dimentional (3D) organotypic coculture model that faithfully represented the histological and biological microenvironment of OC metastasis, to evaluate the effect of SNAI2 upregulation on the ability of CAFs to affect tumor cell growth. Our results demonstrated that SNAI2 upregulation in fibroblasts increased their ability to support the growth of cocultured OC cells (Fig. 4e).

Conversely, silencing of SNAI2 in MRC5-CAFs and primary CAFs resulted in the sharp reduction of the aforementioned typical CAF markers (Additional file 7: Figure S6A). In addition, F-actin staining revealed that SNAI2 knockdown inhibited the spreading of the activated form of MRC5-CAFs and primary CAFs (Additional file 7: Figure S6B–C). Moreover, collagen contraction assay implied that activated fibroblasts displayed a remarkably decreased capacity in contracting ECM after the attenuation of SNAI2 expression (Additional file 7: Figure S6D). Ultimately, SNAI2 silencing in activated fibroblast diminished their role in supporting tumor cell growth in 3D organotypic coculture model (Additional file 7: Figure S6E). Thus, we demonstrated here that SNAI2 reprogram normal quiescent fibroblasts into a proinvasive phenotype that fuels tumor cell growth and invasion.

### SNAI2 drives a transcriptional program in mesenchymal OC that predicts clinical outcome

**Fig. 5:**
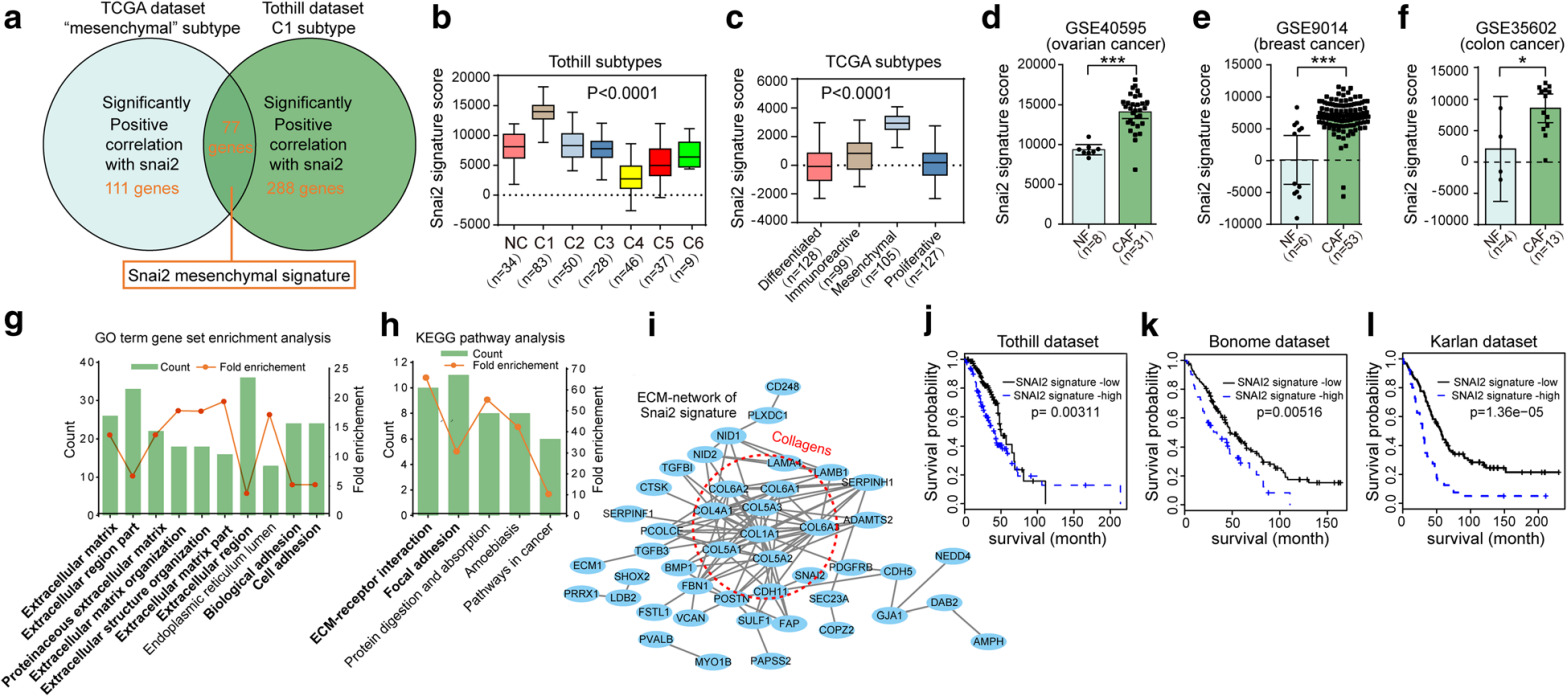
SNAI2 Drives a Transcriptional Program in the mesenchymal OC subtype that predicts clinical outcome. **a** Graphical depiction of the designation of the SNAI2 mesenchymal signature through overlapping genes significantly correlated with snai2 expression in the TCGA mesenchymal and Tothill C1 subtype. **b** and **c** Single sample GSEA (ssGSEA) analysis showing the relative expression of the SNAI2 mesenchymal signature score in various subtypes included in the Tothill’s dataset (**b**) and the ovarian TCGA dataset (**c**). **d–f** ssGSEA analysis showing the relative expression of the SNAI2 mesenchymal signature in NFs and CAFs in the stroma profile of OC (GSE0595) (**d**), breast cancer (GSE9014) (**e**) and colon cancer (GSE35602) (**f**). **g** and **h** Top ten most enriched GO annotation (**g**) and five most enriched KEGG pathways (**h**) in the set of the 77 genes included in SNAI2 mesenchymal signature. Count: number of genes that are involved in a given pathway. The fold enrichment shows how many fold more a given term was overrepresented in the 77 genes compared with a background of the total human genome. **i** STRING (Search Tool for the Retrieval of Interacting Genes) analysis of the proteins included in the SNAI2 mesenchymal signature, with the ECM network central in the network. Lines represent associations between proteins, and the line thickness reflects the number lines of evidence for each association. The dashed circles indicate the proteins included in the collagen family. **j–l** Kaplan-Meier analysis of the SNAI2 mesenchymal signature score expression in the patients included in three independent sets of the OC as a Tothill’s dataset (GSE9891) (**j**), Bonome’s dataset (GSE26712) (**k**) and Karlan’s dataset (GSE51088) (**l**). Kaplan-Meier curves were performed using the log-rank test. (*P < 0.05, ***P < 0.001)

Based on the above findings, we tried to investigate the molecular mechanism that SNAI2 regulates CAF activity. In view of our aforementioned observation that SNAI2 was particularly expressed by stromal fibroblasts in the OC subtype with fibrotic stroma, we therefore explored the SNAI2 regulated signature genes in the TCGA mesenchymal subtype and the Tothill C1 subtype samples. We identified a cluster of 111 genes (Additional file 1: Table S4) that were notably positively correlated with SNAI2 in the TCGA mesenchymal subtype, of which the fibroblasts were demonstrated to be the major constituent [21]. Overlapping the calculated 288 genes (Additional file 1: Table S5) that were significantly positively correlated with SNAI2 in the Tothill C1 subtype, we identified a 77-gene mesenchymal signature SNAI2 defined in the desmoplasia OC subtype (Additional file 1: Table S6) (Fig. 5a). Subsequent qPCR analysis confirmed that SNAI2 positively regulated the expression of the genes included in our calculated “SNAI2 mesenchymal signature” (Additional file 8: Figure S7). Indeed, our defined signature was more prone to overlap with a “stromal signature” rather than an “epithelial signature,” an “activated stromal signature” rather than a “normal stromal signature,” a “141-stromal signature” rather than a “141-immune signature,” suggesting that the SNAI2 mesenchymal signature represents CAFs (Additional file 9: Figure S8A–C). To exclude the possibility that the SNAI2 correlated gene set represents the immune cells in the tumor microenvironment, we performed data mining and revealed that the gene set was highly represented in fibroblasts but not in hematopoietic cell lineages (Additional file 9: Figure S8D).

To further characterize our defined SNAI2 mesenchymal signature, we confirmed in the TCGA and Tothill datasets that the SNAI2 signature score was discriminately expressed in different clusters, and it was most obvious in the desmoplasia subtype (Fig. 5b–c). In addition, the SNAI2 signature score was notably elevated in the CAFs compared with their normal counterparts in the stromal profiles of ovarian, breast, and colon cancer (Fig. 5d–f). Furthermore, a functional analysis of the 77 genes included in the signature revealed their aggregation in cellular function related with ECM remodeling (Fig. 5g–h). A subsequent signal-net analysis revealed that the collagen family members were enriched in the signature, implying that ECM molecule deposition and remodeling played a pivotal role concerning SNAI2 function in fibroblasts (Fig. 5i). Finally, our defined SNAI2 regulated signature predicted patient survival in the Tothill, Bonome, and ovarian TCGA datasets (Fig. 5j–l).

Attempt was made to interpret how SNAI2 transformation of normal fibroblasts. Our preliminary data showed that SNAI2 was closely related with the TGFβ1 signaling in fibroblasts through integrative analysis of ovarian stromal profile and C1 subtype ovarian tumoral profile (Additional file 10: Figure S9A–D). Attenuation of SNAI2 inhibited TGFβ1 activation of downstream signaling in normal fibroblasts and its acquisition of CAF markers (Additional file 10: Figure S9E).

### Matrix stiffening modulates fibroblast activation involving upregulating SNAI2 expression

**Fig. 6:**
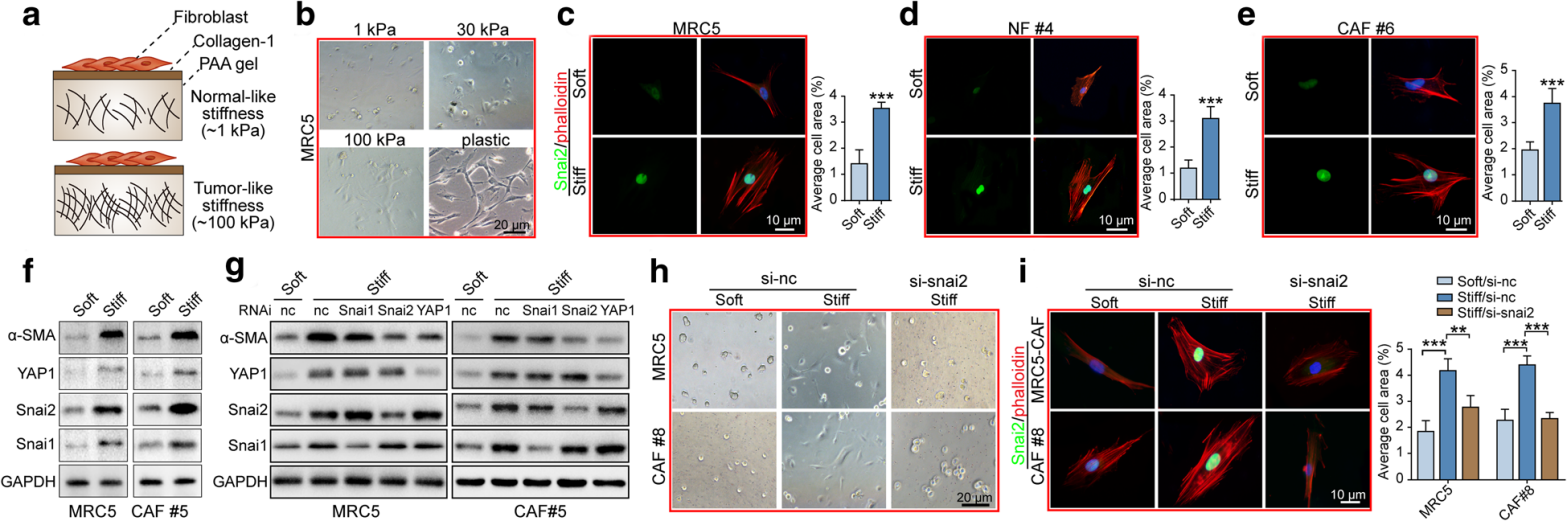
Matrix stiffening modulates fibroblast activation involving upregulating SNAI2 expression. **a** Schematic illustration of the generation of normal-like stiffness (~1kpa) and tumor-like stiffness (~100 kpa) using the PAA gel substrate. **b** Representative images of the MRC5 cells growing on substrate with variant matrix stiffness (1~100 kpa), with a plastic plate being a positive control for cell spreading. **c–e** Representative images and quantification of the cellular cytoskeleton by F-actin staining in the MRC5 cells (**c**), the primary NFs (**d**) and the CAFs (**e**) grown on a soft or stiff matrix. **f** Western blot showing the protein expression of ACTA2, YAP1, SNAI2 and SNAI1 in the MRC5 cells and the primary CAFs grown on a soft or stiff matrix. **g** Immunoblotting showing the protein expression of ACTA2, YAP1, SNAI2 and SNAI1 in the MRC5 cells and the primary CAFs grown on soft or stiff matrix, in the presence or absence of a scramble siRNA or an siRNA specifically targeting YAP1, SNAI2 or SNAI1. **h** Representative images of the MRC5 cells and the primary CAFs grown on a soft or stiff substrate and in the presence or absence of an siRNA specifically targeting SNAI2. **i** Representative images and quantification of the cellular cytoskeleton by F-actin staining in MRC5 cells and in primary CAFs grown on a soft or stiff substrate and in the presence or absence of an siRNA specifically targeting SNAI2

In accordance with the recognition that ECM is largely synthesized and modulated by stromal fibroblasts [31], we showed here that SNAI2 drove a mesenchymal signature and contributed to ECM remodeling. Increased collagen deposition in the ECM leads to increased tissue stiffness, which feeds back to facilitate tumor metastasis [32]. Whether these mechanical signals in turn influence CAF function and how this occurs is only just being appreciated. A COLI-coated polyacrylamide (PAA) gel with different stiffness substrates (1~100 kPa) was adopted to examine the effect of stromal stiffness on fibroblasts (Fig. 6a). We observed a matrix stiffness grade-dependent increase in the spreading of MRC5 cells grown on the PAA gel with a gradient stiffness (Fig. 6b). Moreover, immunofluorescence showed that a stiff matrix significantly upregulated SNAI2 expression and facilitated the spreading of cellular cytoskeleton in MRC5 cells, primary NFs and CAFs (Fig. 6c–e). In accordance with previous reports that YAP signaling is required for mechanotransduction in CAFs [33], a stiff matrix increased the protein level of aSMA, YAP1, SNAI1, and SNAI2 in MRC5 cells and primary CAFs (Fig. 6f).

To better understand the involvement of SNAI2 in the regulation of CAF activation exposed to a stiff matrix, we used RNA interference to selectively attenuate the expression of the aforementioned TFs upregulated by stromal stiffness. Strikingly, the silencing of YAP1 and SNAI2, but not SNAI1, hampered aSMA increase in fibroblasts exposed to a stiff substrate, implying the critical role of SNAI2 in response to a stiff matrix of fibroblasts (Fig. 6g). Similarly, the attenuation of SNAI2 inhibited the fibroblast spreading and cytoskeleton transformation in response to stiff matrix (Fig. 6h–i). These data indicated that SNAI2 reprogrammed fibroblasts to induce a stiff matrix, providing a feed-forward mechanism that increased SNAI2 expression in stromal fibroblasts to sustain their constitutive activation.

### SNAI2 is required for the procarcinogenic role of fibroblast in OC xenograft model

**Fig. 7:**
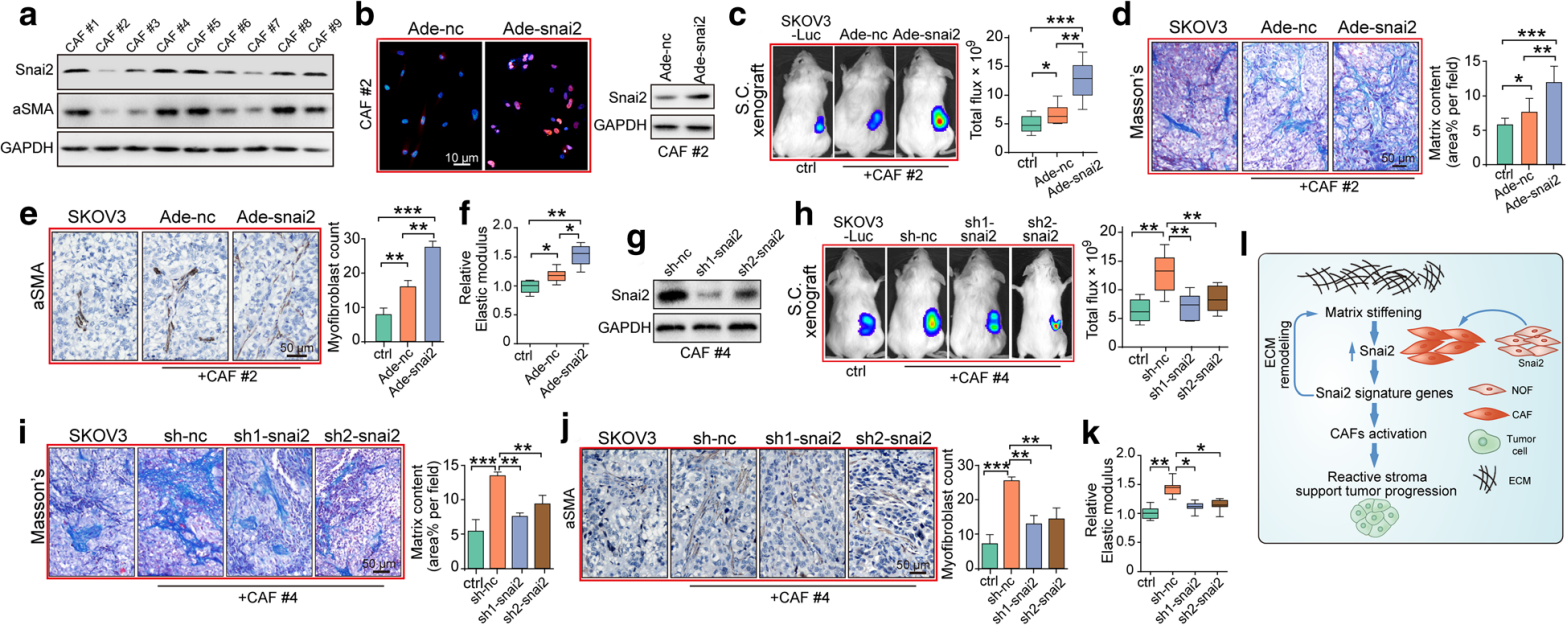
SNAI2 is required for the procarcinogenic role of fibroblasts in an OC xenograft model. **a** Western blot showing the protein expression of SNAI2 and aSMA in a panel of primary CAFs isolated from metastatic tumor tissues of serous OC patients. **b** Left: Immunofluorescence staining of SNAI2 to verify the acquisition of SNAI2 expression in primary CAFs after Ade-SNAI2 transfection (left panel). Right: Western blot analysis confirmed the effect of the SNAI2 expressing adenovirus in increasing SNAI2 expression. **c** Representative bioluminescence images of mice (*n* = 8 each group) bearing SKOV3-Luc alone or co-injection with primary CAFs transfected with either Ade-NC or Ade-SNAI2 at 4 weeks after tumor implantation. Bar graph showing the quantification of the normalized total photon counts of the subcutaneous xenografts in each group. **d** Masson trichrome staining and quantification of the matrix content in tumor sections from mice in groups described in (**c**). **e** Representative aSMA staining images and quantification of the amount of myofibroblasts in xenograft tissues of the above groups. **f** Xenograft tissue stiffness was measured as Young’s Modulus and presented as the relative elastic modulus compared with that of the SKOV3 alone group. **g** Western blot analysis confirmed the silencing efficiency of sh-SNAI2 in reducing SNAI2 expression in the primary CAFs. **h** Representative bioluminescence images of mice (n = 8 each group) bearing SKOV3-Luc alone or co-injection with primary CAFs transfected with either sh-NC or sh-SNAI2 at 4 weeks after tumor implantation. Bar graph showing the quantification of the normalized total photon counts of the subcutaneous xenografts of each group. **i** Masson trichrome staining and quantification of the matrix content in tumor sections from mice in groups described in (**h**). **j** Representative aSMA staining images and quantification of the amount of myofibroblasts in xenograft tissues of the above groups. **k** Xenograft tissue stiffness was measured as Young’s Modulus and presented as the relative elastic modulus compared with that of the SKOV3 alone group. **l** Graphical illustration of the feed-forward regulation mechanism between matrix stiffness and SNAI2 to drive CAF activation, desmoplasia and tumor progression. Matrix stiffness promotes SNAI2 expression in stromal CAFs, which feeds back to maintain a stiff matrix and the subsequent SNAI2 mesenchymal signature that drives a reactive tumor stroma and facilitates tumor promotion. (*P < 0.05;**P < 0.01; ***P < 0.001)

To further explore whether SNAI2 expression in stromal fibroblasts plays a role in supporting OC cells in vivo, we purified several primary fibroblasts from OC tissues. Immunoblotting revealed that SNAI2 was discriminately expressed in those primary CAFs and that SNAI2 expression was positively correlated with aSMA level (Fig. 7a). In the SNAI2-low expressing fibroblast cells (CAF#2), we used an adenovirus encoding SNAI2 to overexpress SNAI2 expression (Fig. 7b). We mixed CAF#2 transfected with either negative control adenovirus (Ade-nc) or SNAI2 expressing adenovirus (Ade-SNAI2) with SKOV3-Luc cells and co-injected them subcutaneously into NOD/SCID mice. Tumors arising from the Ade-SNAI2 CAFs co-injection group grew significantly faster than those in the Ade-nc CAFs co-injection group, and both groups developed larger tumors than the SKOV3-Luc solitary group (Fig. 7c). At the end of the experiment, tumor tissues were subjected to Masson’s trichrome staining and immunological evaluation of aSMA, showing that the matrix content in tumors of the Ade-SNAI2 co-injection group was the strongest, followed by the Ade-nc co-injection group and the SKOV3-Luc solitary group (Fig. 7d–e). Correspondingly, tumor stiffness in xenografts developed in the Ade-SNAI2 co-injection group was the strongest (Fig. 7f).

In another parallel experiment concerning the SNAI2-high expressing fibroblast cells (CAF#4), we used lentiviruses containing the SNAI2-targeting sequence to attenuate SNAI2 expression. Immunoblotting confirmed the stable knock down efficiency of the lentiviruses in silencing the fibroblast SNAI2 level (Fig. 7g). Accordingly, tumors arising from the SKOV3-Luc cells co-injected with sh-SNAI2 CAFs grew significantly slower than those co-injected with the sh-nc CAFs, and both of the groups developed larger tumors than the SKOV3-Luc solitary group (Fig. 7h). Finally, Masson’s trichrome, aSMA staining and tumor stiffness examination revealed that stroma desmoplasia degree of the tumors in the sh-SNAI2 co-injection group was notably lower compared with the sh-nc co-injection group, and both were more pronounced than the SKOV3-Luc solitary group (Fig. 7i–k). Taken together, our data showed here that SNAI2 expression supported the emergence of a CAF-like cell state, impacted tumor matrix stiffness and in turn regulated fibroblast SNAI2 expression and its subsequent activation (Fig. 7l).

## Discussion

A majority of studies on SNAI2 have focused on its role in tumor cells. In this study, we extended the study of SNAI2 from tumor cells to stromal fibroblasts and discovered that SNAI2 and its associated mesenchymal signature were enriched in the desmoplasia subtype of OC. Moreover, SNAI2 and its defined signature were strongly correlated with tumor stroma activation and OC patient survival. Furthermore, the increased ECM stiffness exerted by SNAI2 in turn able to elevate fibroblast SNAI2 expression and sustain fibroblast activation. Together, our work establishes a role for stromal SNAI2 exertion on fibroblast activation and the facilitation of OC advancement, suggesting that SNAI2 could be a target of choice in aiming the stromal constituent of OC.

The importance of tumor stroma has long been appreciated and it is now widely accepted that CAFs exhibit multiple functions that fuel tumor metastasis [34]. Molecular profiling has described a subset of the OC samples characterized by an extensive stromal desmoplasia and associated with the poorest survival. Accumulating evidence indicates that CAFs activity perturbs not only the biochemical but also the biomechanical homeostasis of the tumor microenvironment, which ultimately affects tumor cell behavior and promotes cancer advancement [35]. These data highlighted that targeting stromal CAFs could alleviate stroma desmoplasia and the resultant disease exacerbation. In this setting, our analysis showed that SNAI2 was elevated specifically in the tumor stroma along OC progression and was capable of transforming normal fibroblasts into CAFs. Intriguingly, SNAI2 was most highly expressed in the desmoplastic subtype of OC and was correlated with stromal activation. Moreover, as a classic TF regulating the EMT program in various tumor cells [36], SNAI2 was supposed to control the cascade of downstream effector gene activation. Thus, SNAI2 was highly accordant with the requirements for being a potent target of choice in aiming for the cooperative stroma.

SNAI2 encodes a zinc-finger protein of the SNAI family of TFs [37]. It is expressed in the dorsal neural tube and drives EMT, which leads to neuralcrest cell migration during embryonic development [38]. SNAI2 amplification or interaction with specific oncogenes has been demonstrated in a wide spectrum of human cancers [39]. It was recently demonstrated that SNAI2 is involved in determining the stem cell state of breast and colon cancer [40, 41]. Accumulating evidence revealed that TF expression and function are likely to be context-dependent, and different cell or tumor types respond distinctly to TF regulation [42]. In this regard, both the functional roles in the tumor and the stroma compartment should be taken into consideration in determining the integrative effect of a particular TF. For instance, another SNAI family member, SNAI1, initiates the EMT program and regulates cancer metastasis in tumor cells. In stromal fibroblasts, SNAI1 regulates paracrine signaling and cell transformation [17]. More recently, Snail1-expressing fibroblasts display mechanical properties that support metastasis in breast cancer [18]. In parallel, Twist1 expression was correlated with fibroblast activation in gastric cancer. In OC, SNAI2 was previously reported to induced EMT and was correlated with tumor metastasis and angiogenesis [15]. In addition, our finding first revealed that SNAI2 particularly exists in fibroblast cells and influences stroma activation. Meanwhile, the distinct expression pattern of SNAI1 and SNAI2 in cancer cells and fibroblasts coincides with the context-dependent role of TFs, for which the expression shift implied a unique role for SNAI1 and SNAI2 in the tumor and stromal compartment, respectively. Our subsequent finding that SNAI2, rather than SNAI1, correlated with disease outcome further supports the potential role of SNAI2 in OC progression. The exact mechanism involved in SNAI2 regulation of fibroblast activation in OC remains to be elucidated by future studies.

To better elucidate the SNAI2 regulating downstream cascade network in stromal fibroblasts of OC, we first defined a 77-gene SNAI2 mesenchymal signature through an integrative analysis of Tothill’s C1 cluster and the OC TCGA mesenchymal subtype, for which signature was enriched in functional categories concerning collagen deposition and ECM remodeling. Our defined mesenchymal signature emphasized the contribution of SNAI2 to stromal stiffness and was further demonstrated to be correlated with poor patient survival in OC patients. Our finding resembled a recently discovered collagen-remodeling signature regulated by TGF-β signaling that was associated with metastasis and poor survival in OC and an identified reactive stroma signature associated with primary chemoresistance and predicted OC outcome [43, 44], which unanimously implied the crucial role of stromal activation in determining the fate of OC patients. Comparatively, our defined mesenchymal signature could better stratify tumor stroma from their normal counterpart in ovarian, breast, and colon microdissected profiles. These preliminary findings concerning SNAI2 in OC tumor stroma is completing our recognition of the role of SNAI2 in OC.

CAF-mediated changes in the physical properties of ECM facilitate tumor cell invasion and metastasis [45], while the function of these mechanical signals on CAFs is only just being evaluated. Here, we showed that SNAI2 protein levels increase and accumulate in the nucleus of ovarian fibroblasts following exposure to a stiff ECM in culture and further sustain the continuous activation of fibroblasts. YAP was recently reported to be required for CAFs to promote matrix stiffening and was indispensible for matrix remodeling of fibroblast activation in breast cancer [33]. In addition, SNAI1 was also found to mediate the mechanical signals that regulate CAF activity through the influence of the YAP level [19]. In addition to the participation of YAP, SNAI2 upregulation was demonstrated to be involved in the regulation of stromal stiffness on fibroblast activation in OC. This observation was in accordance with the notion of the context-dependent regulation of TFs and implied a crucial role for SNAI2 positive feedback in amplifying the role of stroma stiffness on the tumor and stromal cells.

## Conclusions

In conclusion, this study established a role for stromal SNAI2 that is distinct but highly complementary to its established role in regulating EMT in OC tumor cells. SNAI2 and its regulated signature were particularly expressed in the desmoplasia subtype of OC, discriminated tumor stroma from their normal counterpart, stratified stroma activation and affected patient outcome. Our observations suggest that the inhibition of SNAI2 and the regulated mesenchymal signature to target stromal activation could be a potential approach in targeting the cooperative tumor stroma of OC.

## Additional files

Additional file 1: Table S1. Listing of the Genes included in the signatures used from MSigDB or published literatures. Listing of the detailed genes included in the aforementioned signatures, all of which used for gene set enrichment analysis (GSEA) or single sample GSEA (ssGSEA) analysis were extracted either from MSigDB (http://software.broadinstitute.org/gsea/msigdb/index.jsp) or published literatures. **Table S2.** Clinicopathological characteristics of our enrolled ovarian cancer patients analysed by immunohistochemistry dichotomised by SNAI2 protein expression levels in carcinoma cells or tumor stromal fibroblasts. The associations between SNAI2 expression level and variant clinicopathological characteristics of ovarian cancer patients were analysed with χ2 test (Fisher’s exact test). **Table S3.** Univariate and multivariate Cox regression analysis of SNAI2 expression level and overall survival of ovarian cancer patients. The prognostic significance of SNAI2 expression and other clinicopathological variables was first assessed by univariate Cox, followed by multivariate Cox regression analysis regarding to the overall survival time in a cohort of 160 ovarian cancer patients. **Table S4.** Genes significantly positively correlated with SNAI2 expression in the ‘mesenchymal’ subtype of ovarian TCGA dataset. List of the 111 genes that were calculated significantly positively related with SNAI2 expression in the ‘mesenchymal’ subtype of ovarian TCGA profile, using the R2 Genomic Analysis and Visualization Platform (http://r2.amc.nl). Genes with a Pearson correlation greater than or equal to 0.35 and a *p* value less than 1% were selected from each cohort. **Table S5.** Genes significantly positively correlated with Snai2 expression in the ‘C1’ subtype of Tothill’s dataset (GSE9891). List of the 288 genes that were calculated strongly positively correlated with SNAI2 expression in the ‘C1’ subtype of Tothill’s dataset (GSE9891), using the R2 Genomic Analysis and Visualization Platform (http://r2.amc.nl). Genes with a Pearson correlation greater than or equal to 0.4 and a *p* value less than 1% were selected from each cohort. **Table S6.** Listing of the 77 Genes included in the calculated ‘Snai2 mesenchymal signature’ of ovarian cancer. Designation of our defined ‘Snai2 mesenchymal signature’ and listing of the 77 genes included in the signature, through Overlapping analysis of the gene set that were calculated positively correlated with SNAI2 expression in the ‘mesenchymal’ subtype of ovarian TCGA profile and the ‘C1’ subtype of Tothill’s dataset (GSE9891). (XLSX 120 kb)

Additional file 2: Figure S1. The clinical relevance of SNAI2 in epithelial ovarian cancer patients. Meta-analysis depicting the forest plot of SNAI2 expression as a univariate predictor of overall survival (OS) (A)**.** and progression-free survival (PFS) (B)**.** using several datasets with the applicable gene expression and survival information from the high grade epithelial ovarian cancer patients. (TIFF 507 kb)

Additional file 3: Figure S2. SNAI1 was not discriminately expressed in ovarian tumor stroma and failed to predict patient outcome in OC patients. **A** Boxplots showing the expression level of SNAI1 in microdissected normal epithelium, normal stroma, tumor epithelium and tumor stroma included in the ovarian profile GSE40595. **B** Illustration of the relative gene expression of SNAI1 in the epithelial, leukocyte, and endothelial cells and in the cancer-associated fibroblasts cell population as deposited in the colon cancer profile GSE39396. **C–D** Meta-analysis depicting the forest plot of SNAI1 expression as a univariate predictor of overall survival (OS) (**C**)**.** and progression-free survival (PFS) (**D**)**.** using several datasets with the applicable gene expression and survival information from high grade epithelial ovarian cancer patients. n.s. indicates no significance. (TIFF 2895 kb)

Additional file 4: Figure S3. SNAI2 mRNA expression is associated with poor survival in multiple cancer types. **A** Survival z-scores in different cancer types associated with the expression of SNAI2 mRNA. Red indicates poor survival and blue indicates good survival. **B** Heatmap illustrating the survival z-scores associated with the mRNA expression of the pivotal EMT regulators in major solid cancers. Red indicates poor survival, and blue indicates good survival. The data were obtained from the PREdiction of Clinical Outcomes from Genomic Profiles (PRECOG) database (precog.stanford.edu). (TIFF 3157 kb)

Additional file 5: Figure S4. SNAI2 mRNA expression is associated with CAF markers and ECM remodeling molecules in multiple cancer types. **A** Spearman’s correlation analysis of SNAI2 and classical CAF markers (ACTA2, FAP and PDGFRB) in ovarian TCGA dataset. **B** Spearman’s correlation analysis of SNAI2 and classical ECM remodeling molecules (LOX, COL1A1 and SPARC) in ovarian TCGA dataset. **C** Spearman’s correlation analysis of SNAI2 and classical CAF markers (ACTA2, FAP and PDGFRB) in the ovarian stromal profile GSE40595. **D** Spearman’s correlation analysis of SNAI2 and classical ECM remodeling molecules (LOX, COL1A1 and SPARC) in the ovarian stromal profile GSE40595. (TIFF 1227 kb)

Additional file 6 **Figure S5.** SNAI2 mRNA expression is associated with CAF markers in multiple cancer types. **A–D** Spearman’s correlation analysis of SNAI2 and classical CAF markers (ACTA2, FAP and PDGFRB) in the TCGA dataset of invasive breast cancer (**A**), colorectal adenocarcinoma (**B**), renal clear cell carcinoma (**C**) and glioblastoma (**D**). (TIFF 3443 kb)

Additional file 7: Figure S6. Attenuation of SNAI2 diminished stromal fibroblasts activation. **A** Western blot analysis of PDGFRB, FAP, ACTA2 and SNAI2 in MRC5-CAFs and primary ovarian CAFs after a 72 h transfection with si-ctrl or si-SNAI2. GAPDH served as the loading control. **B** and **C** Representative images and quantification of the cellular cytoskeleton by F-actin staining in MRC5-CAFs (**B**) and primary ovarian CAFs (**C**) in the si-ctrl or si-SNAI2 transfection group. **D** Representative images and quantification of the collagen contraction capacity of the MRC5-CAFs and primary ovarian CAFs in the si-ctrl or si-SNAI2 transfection group. Representative images and the quantification of the proliferation index of SKOV3 cells cocultured with MRC5-CAFs and primary ovarian CAFs in the si-ctrl or si-SNAI2 transfection group (**P < 0.01, ***P < 0.001). (TIFF 4142 kb)

Additional file 8: Figure S7. Validation of SNAI2 regulation of gene expression included in the “snai2 mesenchymal signature”. **A** and **B** qPCR analysis of the relative gene expression of representative genes included in the “snai2 mesenchymal signature” as SNAI2, ADAM12, BMP1, COL1A1, COL5A2, CTSK, FBN1, LAMB1, LAMA4, MARCKS, NID1, POSTN, RCAN2, VCAN, WISP1 in MRC5-CAFs (**A**) and primary ovarian CAFs (**B**) in the si-ctrl or si-SNAI2 transfection group (*P < 0.05, **P < 0.01, ***P < 0.001). (TIFF 1674 kb)

Additional file 9: Figure S8. SNAI2 mesenchymal signature is representative of stromal fibroblast activation. **A** Overlapping analysis of the SNAI2 mesenchymal signature with that of the selected signatures representative of the epithelial or stromal activation in Yeung’s profiles. **B** Overlapping analysis of the SNAI2 mesenchymal signature with that of the “activated stromal signature” and the “normal stromal signature” in Moffitt’s profile. **C** Overlapping analysis of the SNAI2 mesenchymal signature with that of the classical “141-stromal signature” and the “141-immune signature” in Yosihara’s profiles. **D** Expression of the SNAI2 mesenchymal signature genes mapped on the transcriptome of individual murine hematopoietic and stromal celltypes in the ImmGene project (immgen.com). The plot was generated using MyGeneset tool (rstats.immgen.org/MyGeneSet). (TIFF 2226 kb)

Additional file 10: Figure S9. SNAI2 was involved in TGFβ1 signaling activation of normal fibroblasts. **A** Listing of f that SNAI2 correlated in ovarian stromal profile GSE40595. **B** Listing of the top ten ranked fibroblast activation signatures that SNAI2 correlated in C1 subtype tumoral profile of GSE9891.**C** Venn diagram showed the common SNAI2 correlated fibroblast activation signatures in GSE40595 and C1 subtype of GSE9891. **D** Network diagram showed the intimate interaction between “SNAI2 signature genes” and “VERRECCHIA_EARLY_RESPONSE_TO_TGFB1”. **E** Immunoblotting of SNAI2, SMAD3, p-SMAD3, aSMA and FAP in si-Ctrl or si-SNAI2 transfected MRC5 fibroblasts, in the absence or presence of TGFβ1. GAPDH served as the loading control. (TIFF 2580 kb)

## Abbreviations

CAFs: Cancer associated fibroblasts
ECM: Extracellular matrix
GSEA: Gene set enrichment analysis
OC: Ovarian cancer
SNAI1: Snail family zinc finger 1
SNAI2: Snail family zinc finger 2
ssGSEA: Single sample gene set enrichment analysis
TCGA: The Cancer Genome Atlas
TFs: Transcriptional factors
Twist1: Twist basic helix-loop-helix transcription factor 1
YAP1: Yes-associated protein 1
