# Supplementary figures and images for: Reprogramming of stromal fibroblasts by SNAI2 contributes to tumor desmoplasia and ovarian cancer progression
Source: Mol Cancer. 2017 Oct 17;16:163. doi: 10.1186/s12943-017-0732-6 (PMC5645935; doi:10.1186/s12943-017-0732-6)

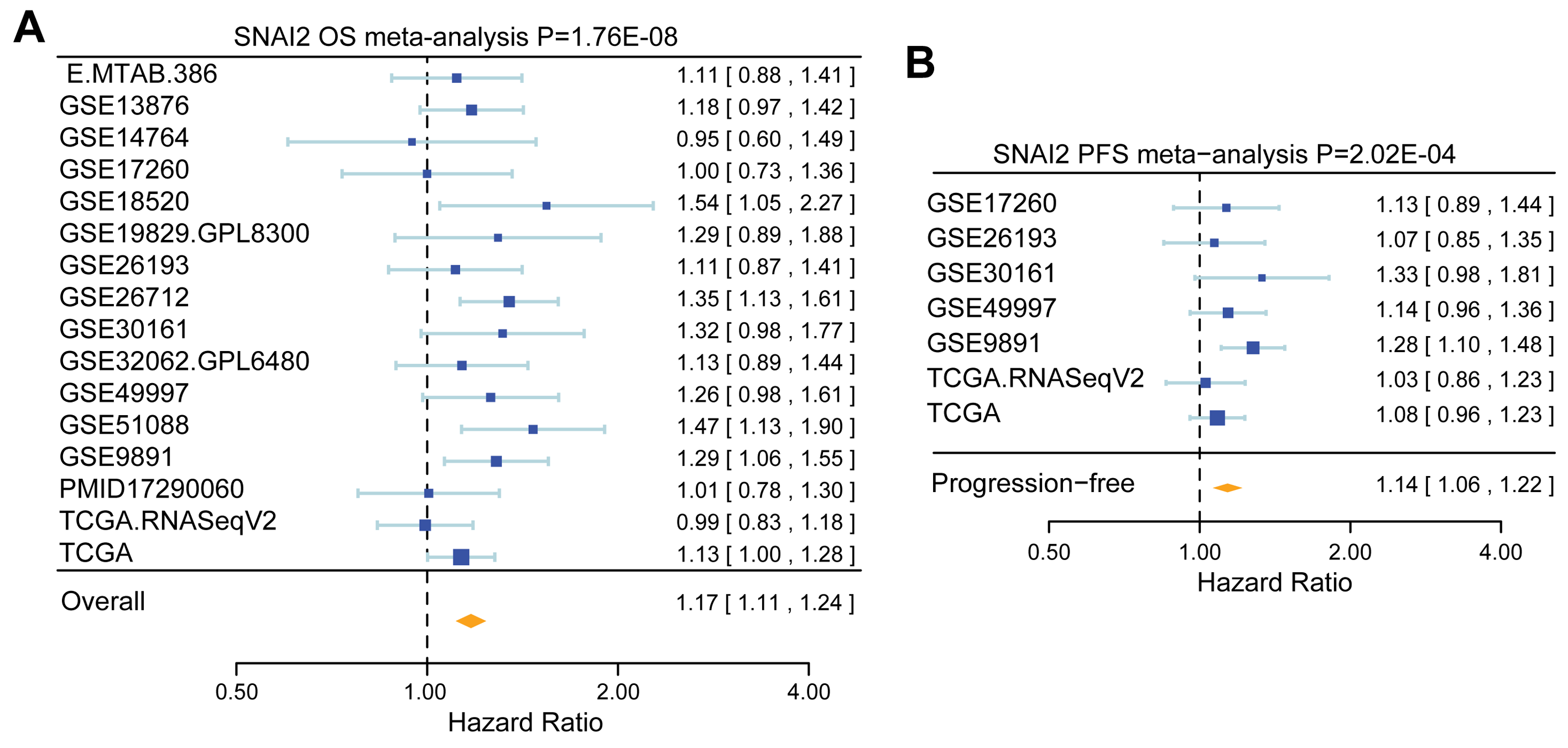

Supplement: Supplementary file 2 — The clinical relevance of SNAI2 in epithelial ovarian cancer patients. Meta-analysis depicting the forest plot of SNAI2 expression as a univariate predictor of overall survival (OS) (A). and progression-free survival (PFS) (B). using several datasets with the applicable gene expression and survival information from the high grade epithelial ovarian cancer patients. (TIFF 507 kb) [file 12943_2017_732_MOESM2_ESM.tif]

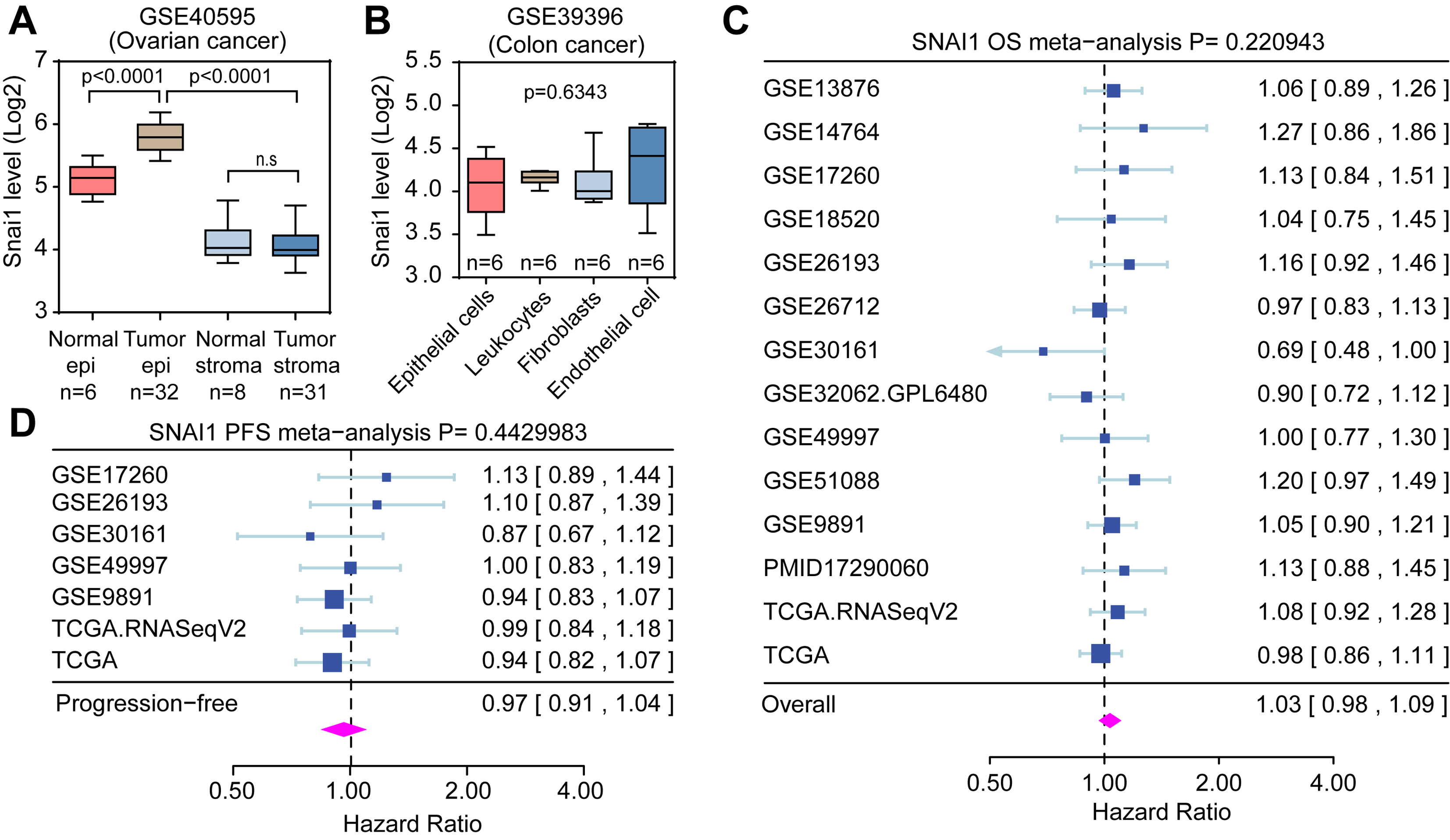

Supplement: Supplementary file 3 — SNAI1 was not discriminately expressed in ovarian tumor stroma and failed to predict patient outcome in OC patients. A Boxplots showing the expression level of SNAI1 in microdissected normal epithelium, normal stroma, tumor epithelium and tumor stroma included in the ovarian profile GSE40595. B Illustration of the relative gene expression of SNAI1 in the epithelial, leukocyte, and endothelial cells and in the cancer-associated fibroblasts cell population as deposited in the colon cancer profile GSE39396. C–D Meta-analysis depicting the forest plot of SNAI1 expression as a univariate predictor of overall survival (OS) (C). and progression-free survival (PFS) (D). using several datasets with the applicable gene expression and survival information from high grade epithelial ovarian cancer patients. n.s. indicates no significance. (TIFF 2895 kb) [file 12943_2017_732_MOESM3_ESM.tif]

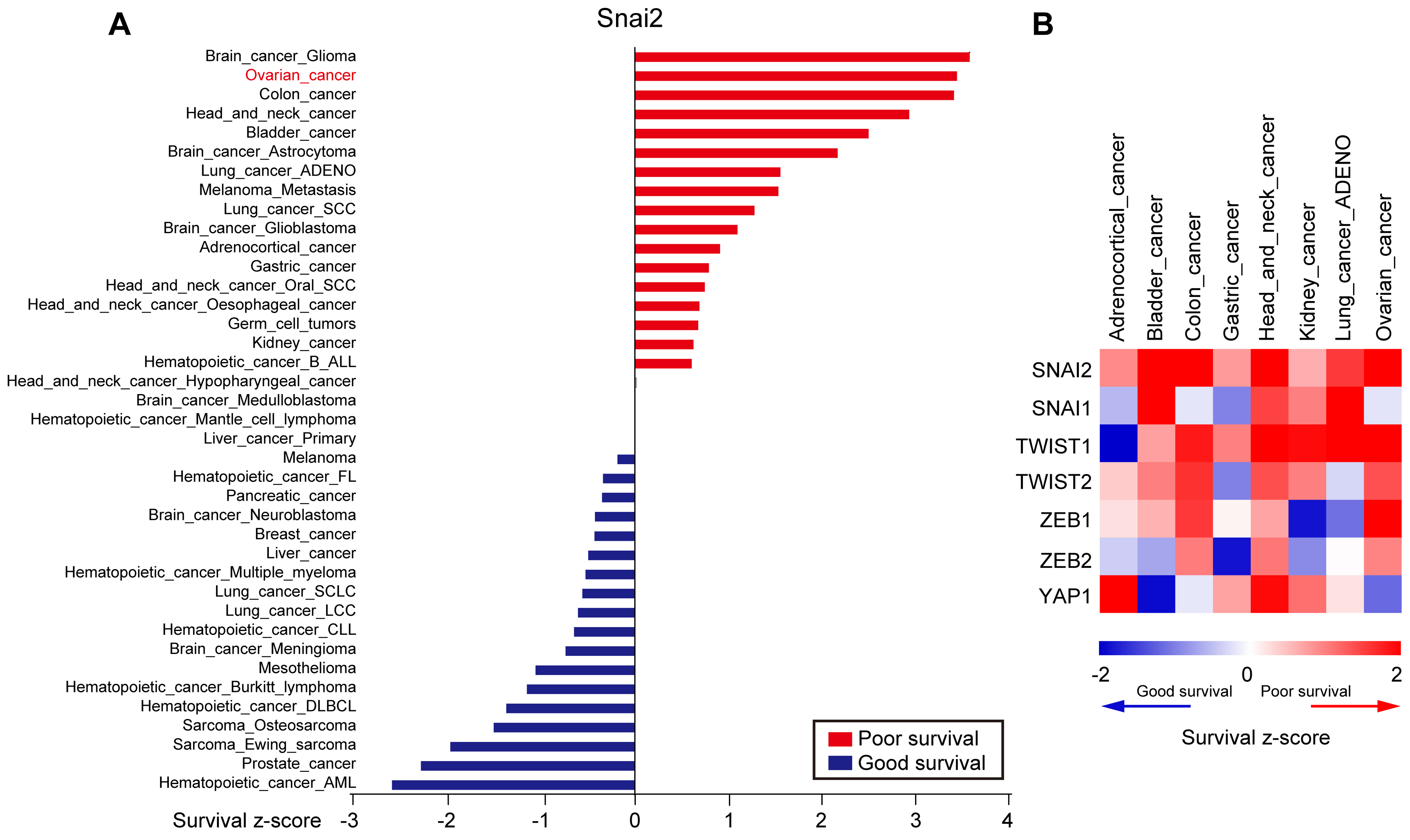

Supplement: Supplementary file 4 — SNAI2 mRNA expression is associated with poor survival in multiple cancer types. A Survival z-scores in different cancer types associated with the expression of SNAI2 mRNA. Red indicates poor survival and blue indicates good survival. B Heatmap illustrating the survival z-scores associated with the mRNA expression of the pivotal EMT regulators in major solid cancers. Red indicates poor survival, and blue indicates good survival. The data were obtained from the PREdiction of Clinical Outcomes from Genomic Profiles (PRECOG) database (precog.stanford.edu). (TIFF 3157 kb) [file 12943_2017_732_MOESM4_ESM.tif]

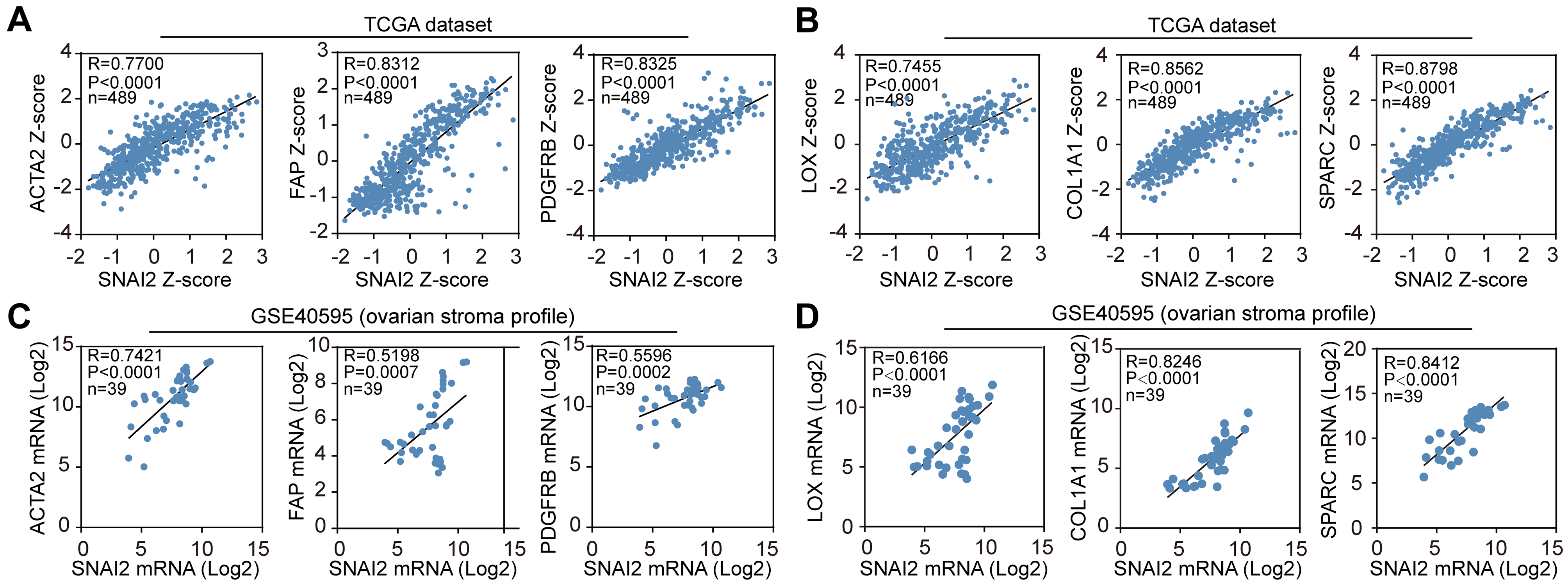

Supplement: Supplementary file 5 — SNAI2 mRNA expression is associated with CAF markers and ECM remodeling molecules in multiple cancer types. A Spearman’s correlation analysis of SNAI2 and classical CAF markers (ACTA2, FAP and PDGFRB) in ovarian TCGA dataset. B Spearman’s correlation analysis of SNAI2 and classical ECM remodeling molecules (LOX, COL1A1 and SPARC) in ovarian TCGA dataset. C Spearman’s correlation analysis of SNAI2 and classical CAF markers (ACTA2, FAP and PDGFRB) in the ovarian stromal profile GSE40595. D Spearman’s correlation analysis of SNAI2 and classical ECM remodeling molecules (LOX, COL1A1 and SPARC) in the ovarian stromal profile GSE40595. (TIFF 1227 kb) [file 12943_2017_732_MOESM5_ESM.tif]

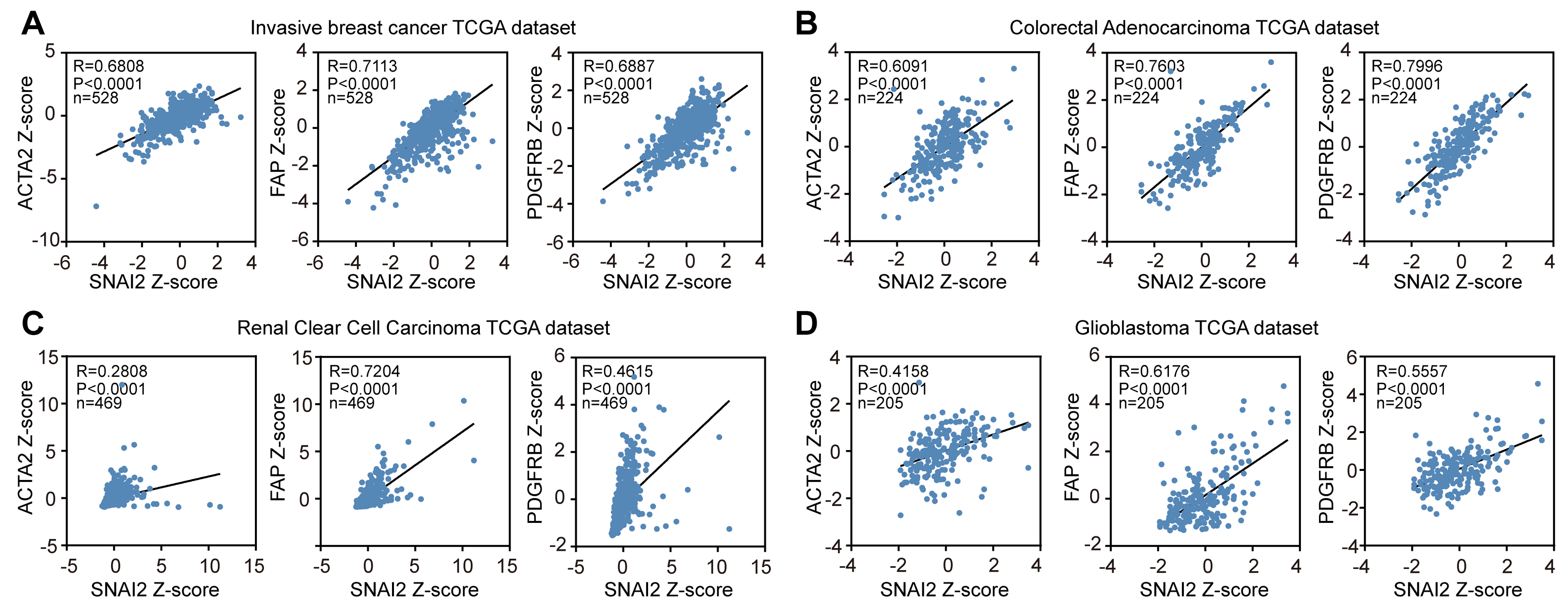

Supplement: Supplementary file 6 — Figure S5. SNAI2 mRNA expression is associated with CAF markers in multiple cancer types. A–D Spearman’s correlation analysis of SNAI2 and classical CAF markers (ACTA2, FAP and PDGFRB) in the TCGA dataset of invasive breast cancer (A), colorectal adenocarcinoma (B), renal clear cell carcinoma (C) and glioblastoma (D). (TIFF 3443 kb) [file 12943_2017_732_MOESM6_ESM.tif]

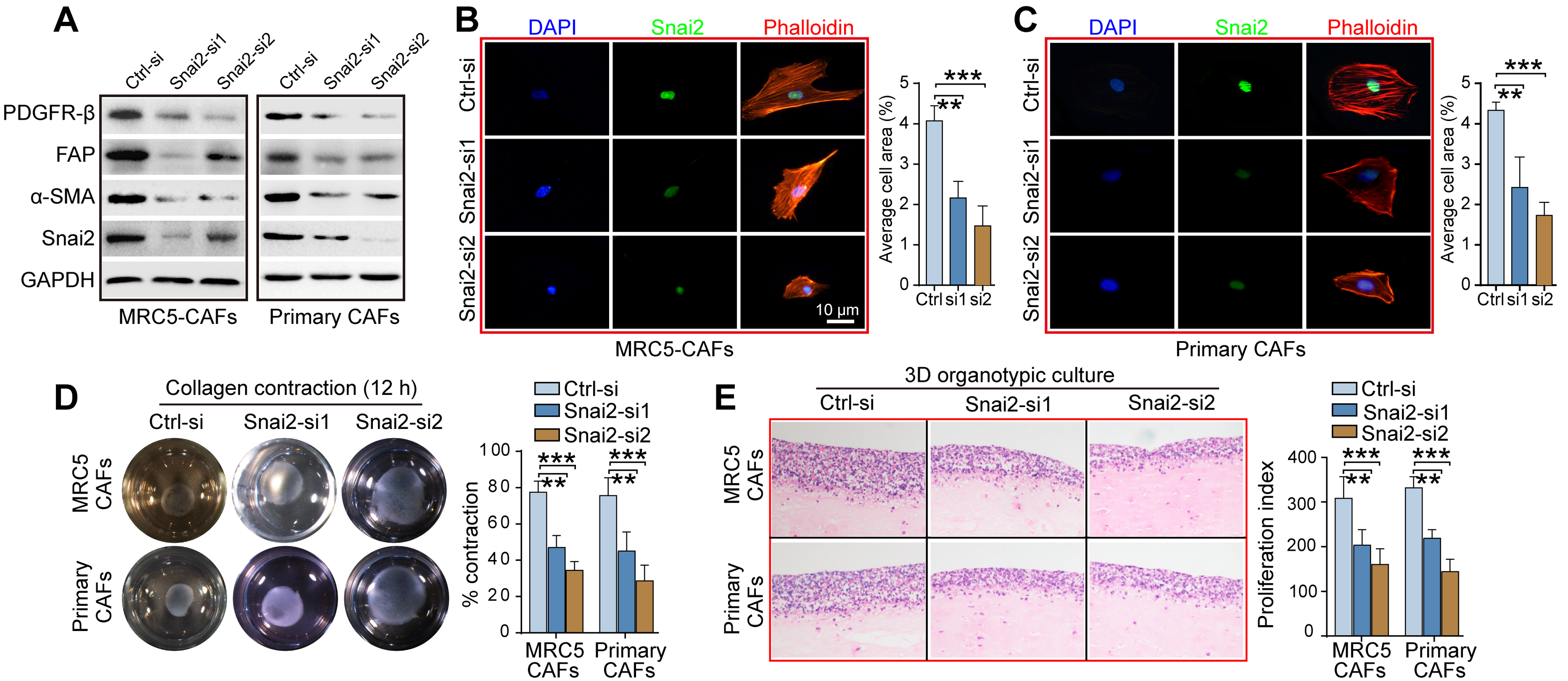

Supplement: Supplementary file 7 — Attenuation of SNAI2 diminished stromal fibroblasts activation. A Western blot analysis of PDGFRB, FAP, ACTA2 and SNAI2 in MRC5-CAFs and primary ovarian CAFs after a 72 h transfection with si-ctrl or si-SNAI2. GAPDH served as the loading control. B and C Representative images and quantification of the cellular cytoskeleton by F-actin staining in MRC5-CAFs (B) and primary ovarian CAFs (C) in the si-ctrl or si-SNAI2 transfection group. D Representative images and quantification of the collagen contraction capacity of the MRC5-CAFs and primary ovarian CAFs in the si-ctrl or si-SNAI2 transfection group. Representative images and the quantification of the proliferation index of SKOV3 cells cocultured with MRC5-CAFs and primary ovarian CAFs in the si-ctrl or si-SNAI2 transfection group (**P < 0.01, ***P < 0.001). (TIFF 4142 kb) [file 12943_2017_732_MOESM7_ESM.tif]

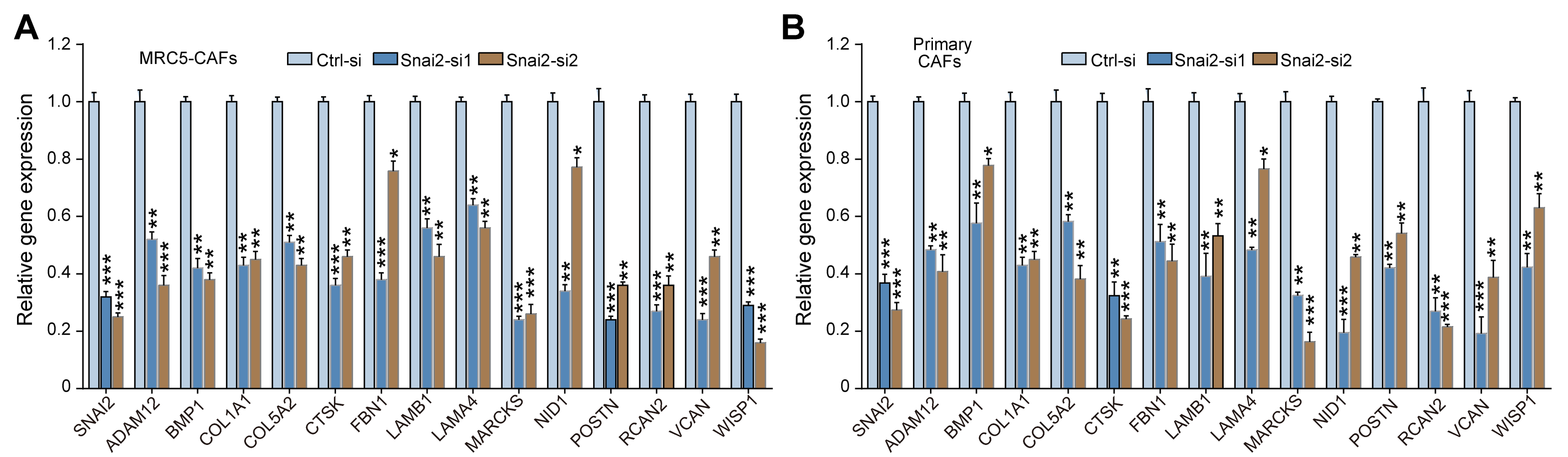

Supplement: Supplementary file 8 — Validation of SNAI2 regulation of gene expression included in the “snai2 mesenchymal signature”. A and B qPCR analysis of the relative gene expression of representative genes included in the “snai2 mesenchymal signature” as SNAI2, ADAM12, BMP1, COL1A1, COL5A2, CTSK, FBN1, LAMB1, LAMA4, MARCKS, NID1, POSTN, RCAN2, VCAN, WISP1 in MRC5-CAFs (A) and primary ovarian CAFs (B) in the si-ctrl or si-SNAI2 transfection group (*P < 0.05, **P < 0.01, ***P < 0.001). (TIFF 1674 kb) [file 12943_2017_732_MOESM8_ESM.tif]

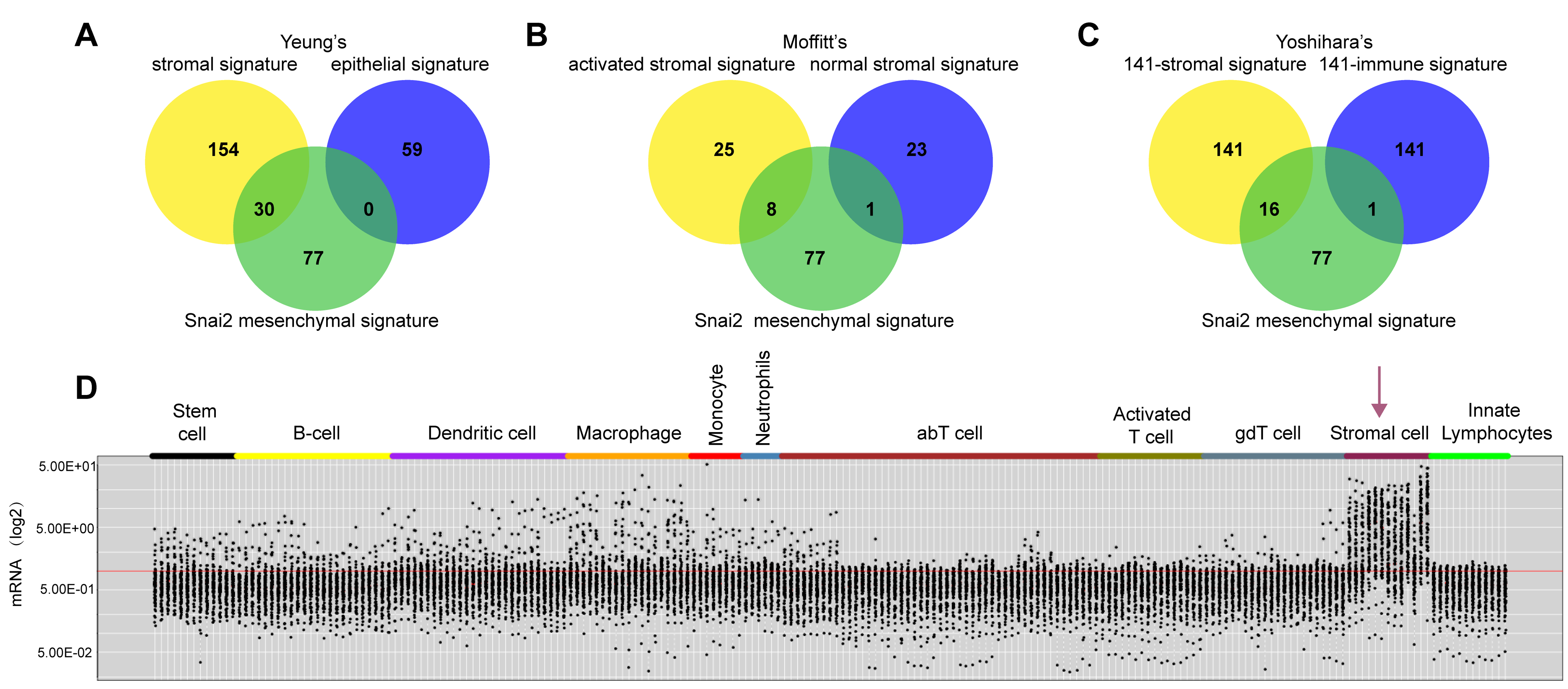

Supplement: Supplementary file 9 — SNAI2 mesenchymal signature is representative of stromal fibroblast activation. A Overlapping analysis of the SNAI2 mesenchymal signature with that of the selected signatures representative of the epithelial or stromal activation in Yeung’s profiles. B Overlapping analysis of the SNAI2 mesenchymal signature with that of the “activated stromal signature” and the “normal stromal signature” in Moffitt’s profile. C Overlapping analysis of the SNAI2 mesenchymal signature with that of the classical “141-stromal signature” and the “141-immune signature” in Yosihara’s profiles. D Expression of the SNAI2 mesenchymal signature genes mapped on the transcriptome of individual murine hematopoietic and stromal celltypes in the ImmGene project (immgen.com). The plot was generated using MyGeneset tool (rstats.immgen.org/MyGeneSet). (TIFF 2226 kb) [file 12943_2017_732_MOESM9_ESM.tif]

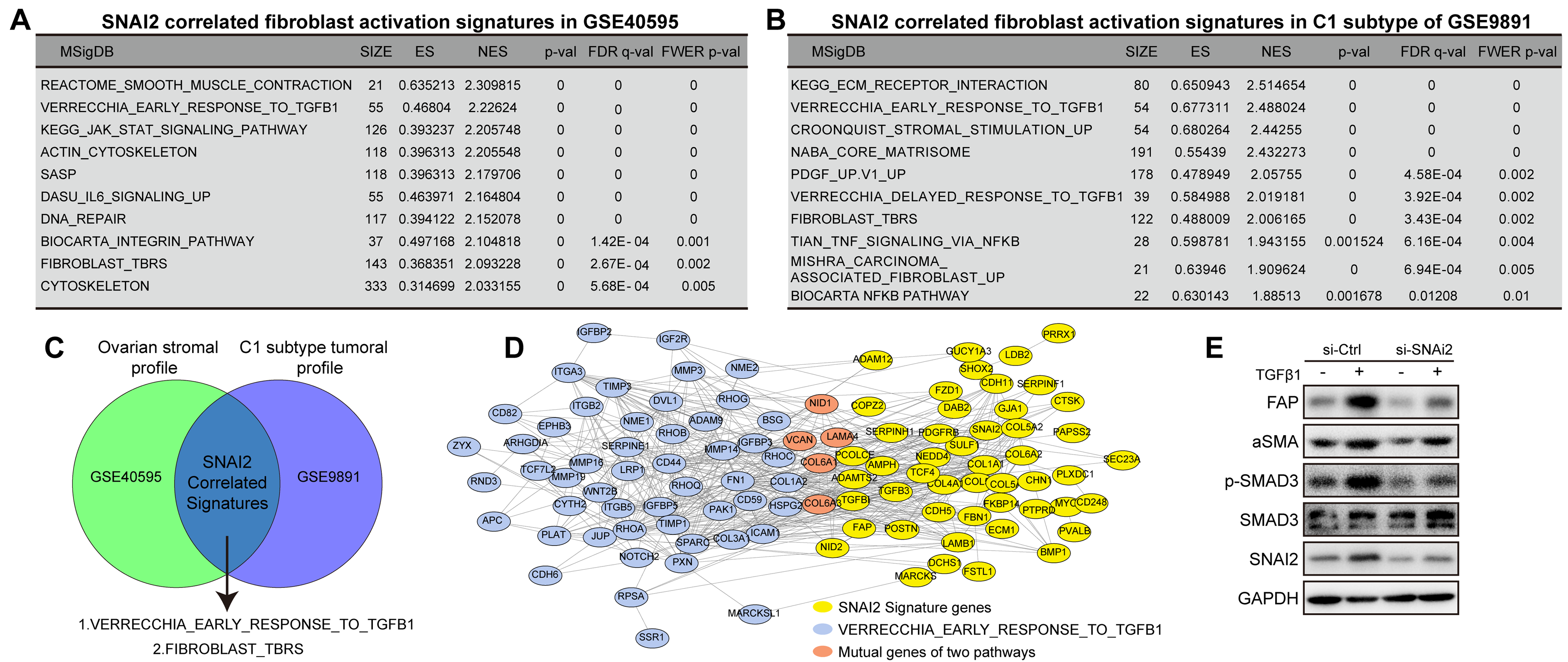

Supplement: Supplementary file 10 — SNAI2 was involved in TGFβ1 signaling activation of normal fibroblasts. A Listing of f that SNAI2 correlated in ovarian stromal profile GSE40595. B Listing of the top ten ranked fibroblast activation signatures that SNAI2 correlated in C1 subtype tumoral profile of GSE9891.C Venn diagram showed the common SNAI2 correlated fibroblast activation signatures in GSE40595 and C1 subtype of GSE9891. D Network diagram showed the intimate interaction between “SNAI2 signature genes” and “VERRECCHIA_EARLY_RESPONSE_TO_TGFB1”. E Immunoblotting of SNAI2, SMAD3, p-SMAD3, aSMA and FAP in si-Ctrl or si-SNAI2 transfected MRC5 fibroblasts, in the absence or presence of TGFβ1. GAPDH served as the loading control. (TIFF 2580 kb) [file 12943_2017_732_MOESM10_ESM.tif]
